# Does Cone-Beam Computed Tomography Provide Additional Benefit to Periodontal Infrabony Defect Topography Assessment? A Multi-Centre Case-Based Questionnaire

**DOI:** 10.3390/dj14090576

**Published:** 2026-09-08

**Authors:** Nicholas David Sung, Tiffany See Nok Chen, Melissa Rachel Fok, Mihai Tarce, Jasmine Loke, Luigi Nibali, Ana Castro, Georgios Pelekos

**Affiliations:** 1 Division of Periodontology and Implant Dentistry, Faculty of Dentistry, The University of Hong Kong, Hong Kong SAR 999077, China; nsung@hku.hk (N.D.S.); tiffc092@connect.hku.hk (T.S.N.C.); melfok@hku.hk (M.R.F.); mihai@hku.hk (M.T.); 2 Section of Periodontology, Department of Oral Health Sciences, KU Leuven and Dentistry, University Hospitals, KU Leuven, 3000 Leuven, Belgium; ana.castro@uzleuven.be; 3 Periodontology Unit, Centre for Host Microbiome Interactions, Faculty of Dentistry, Oral & Craniofacial Sciences, King’s College London, London SE1 1UL, UK; jasmine.h.loke@kcl.ac.uk (J.L.); luigi.nibali@kcl.ac.uk (L.N.)

**Keywords:** CBCT, periodontal diagnostics, periodontal education, infrabony defect

## Abstract

**Objectives:** The objectives of this study were to investigate whether cone-beam computed tomography (CBCT) provides additional benefit in assessing periodontal infrabony defect topography, and whether assessment accuracy differed between clinicians in periodontology with different levels of experience. **Background:** A comprehensive assessment of the periodontal condition usually comprises clinical periodontal examination and two-dimensional radiographic evaluation. Whilst periapical radiographs are the most frequently employed for radiographic assessment of periodontal bone loss, they have some limitations in assessing infrabony defects. As a result, CBCT has been gaining attention recently as an adjunctive periodontal diagnostic modality that can overcome these limitations. It is well established that the use of CBCT for periodontal diagnosis is not routine and should only be reserved for judicious case selection in accordance with the as low as reasonably achievable (ALARA) principle. Given these considerations, studies evaluating whether CBCT provides any additional benefit in the assessment of periodontal infrabony defects remain limited, and no formal guidelines currently exist regarding its role or implementation in postgraduate periodontal education. **Methods:** Forty-six participants (26 residents and 20 specialists in periodontology) from three European Federation of Periodontology (EFP)-accredited centres were invited: King’s College London (KCL), United Kingdom; KU Leuven, Belgium; and The University of Hong Kong (HKU), Hong Kong SAR, China. The participants were required to complete two case-based questionnaires on infrabony defect assessment, with a two-week washout period in between. Eight complex cases were presented, once with periodontal charting and periapical radiography (Questionnaire S1), and once with CBCT images in addition to the conventional data (Questionnaire S2). For all eight cases, an HKU panel performed reference-standard intra-surgical or CBCT measurements against which the participants’ answers were scored. **Results:** Forty participants returned the questionnaires (25 residents and 15 specialists). The response rate was, therefore, 87.0%. Participants assessed the infrabony defects more accurately in Questionnaire S2 compared with Questionnaire S1 (the mean scores [%] were 69.8 ± 11.9 and 63.5 ± 11.8 respectively); the mean difference was statistically significant (*p* < 0.001). When comparing residents and specialists, the mean difference in scores (%) was 0.5 (95% CI: −7.4–8.5) for Questionnaire S1 and −2.5 (95% CI: −10.4–5.5) for Questionnaire S2, with neither difference reaching statistical significance (*p* > 0.05). However, a larger study is required to draw more definitive conclusions about differences between clinician groups. **Conclusions:** CBCT, adjunctive to conventional periodontal diagnostics, provided statistically significant additional benefit in assessing infrabony defect topography by increasing assessment accuracy. However, whether the magnitude of this improvement is clinically meaningful requires further research. This study shows that between residents and specialists in periodontology, there was no statistically significant difference in the accuracy of infrabony defect topography assessment; nonetheless, given the limited sample size, further work is needed to clarify potential between-group differences. Whilst keeping the ALARA principle in mind, this study highlights the need for further investigation into the value of CBCT in complex periodontal cases, emphasises the importance of defining its role in postgraduate periodontal education, and underscores the need for a larger-scale study involving additional EFP-accredited centres to enable more robust conclusions.

## 1. Introduction

A comprehensive assessment of the periodontal condition is required to establish an accurate diagnosis of periodontal disease and derive the most appropriate treatment plan [1]. To achieve this, a precise evaluation of both the soft and hard tissues comprising the periodontal apparatus is necessary. Multiple periodontal parameters recorded during clinical examination contribute to soft-tissue assessment. Radiographic evaluation, in the form of intra-oral radiographs, assesses the hard tissues by identification and quantification of horizontal, vertical and interradicular bone loss.

Intra-oral periapical radiographs remain the most employed modality for radiographic assessment of periodontal structures due to their accessibility, affordability, and high resolution [2]. Current evidence also supports their role as the gold standard for comprehensive periodontal diagnosis. However, this modality comes with certain limitations. In terms of defect visibility, certain anatomical structures could mask an infrabony defect and make it radiographically invisible [3]. In terms of defect depth, linear radiographic measurements were reported to be poor estimates of the actual extent of bone loss in infrabony defects [4]. In terms of defect topography, since periapical radiographs yield two-dimensional renderings of three-dimensional structures, information on defect topography (such as defect extent and configuration) may not be clearly reflected [5].

To overcome the limitations associated with two-dimensional intra-oral radiography, there is increasing attention being paid to investigating the usefulness of cone-beam computed tomography (CBCT) in clinical periodontal practice [6]. As an adjunctive diagnostic method, CBCT provides measurements free from distortion and magnification and permits assessment of periodontal bone loss without overlap of anatomical structures. It also provides three-dimensional information for a more accurate evaluation of periodontal osseous defects, such as in the case of infrabony defects and furcation defects [2,7,8,9]. In infrabony defects, the agreement of CBCT with direct intra-surgical measurements regarding defect type and extension appeared to surpass that of measurements on a periapical radiograph [10]. Whilst the principle of keeping radiation exposure as low as reasonably achievable (ALARA) must always be upheld, in some complex case scenarios, the information that CBCT provides may offer valuable insight to the clinician when assigning periodontal diagnosis and prognosis on a tooth-by-tooth basis and when formulating a periodontal treatment plan [11].

The available evidence suggests that CBCT may have a selective role in contemporary periodontal practice. Suphanantachat and coworkers proposed that CBCT was a valuable treatment decision-making aid for periodontal regeneration and tooth extraction [12]. In 2017, a review by the American Academy of Periodontology suggested that although there is little evidence for CBCT as a routine replacement or adjunct to 2D imaging in managing periodontitis, CBCT may be a valuable addition to clinical periodontal examination and two-dimensional radiographs in selected patients with advanced periodontal disease [13]. Examples included in the review were clinical scenarios in which three-dimensional imaging may (1) confirm assignment of teeth with hopeless periodontal prognosis, which improves treatment decision making, and (2) improve pre-surgical assessment of periodontal osseous defect topography, which, in turn, may influence the surgical flap design, especially when minimally invasive surgical approaches are considered. More recently, in the ‘Consensus Report of the 20th European Workshop on Periodontology: Contemporary and Emerging Technologies in Periodontal Diagnosis’, the authors concluded that because of exposure to ionising radiation and from cost–benefit considerations, routine use of CBCT for periodontal diagnosis is not advised. However, in specific circumstances involving the presence of infrabony and furcation defects, CBCT imaging may provide additional detail to 2D radiographic imaging, which may benefit diagnosis, decision making and treatment planning [14]. For example, after Steps 1 and 2 of periodontal therapy, if endpoints of therapy are not fulfilled, additional site-specific 3D imaging assessment can be considered. Similarly, in the 2026 clinical recommendations by the American Dental Association and American Academy of Oral and Maxillofacial Radiology for patient selection for dental radiography and cone-beam computed tomography, it was recommended that the management of periodontal disease, in general, does not warrant use of CBCT, although CBCT may be indicated for treatment planning of complex cases [15]. These conclusions imply that CBCT use in periodontitis management should be judicious and case-dependent, reserved for cases involving more complex periodontal osseous defects.

Despite the increasing available evidence, to date, there is still both a clinical and educational gap to address. Clinically, studies investigating whether CBCT offers any additional benefit in periodontal infrabony defect assessment and treatment planning are limited. This study aims to address this clinical gap by investigating if CBCT provides additional benefit in assessing periodontal infrabony defect topography as well as in Step 3 periodontal treatment planning for complex cases, when used as an adjunct to conventional periodontal diagnostics (periodontal charting and periapical radiography). To explore the value of CBCT in periodontology for clinicians with different levels of experience, this study also aims to compare infrabony defect assessment accuracy, with and without CBCT, between postgraduate residents and specialists in periodontology. Educationally, there remains scope for research into the application of CBCT in periodontal treatment planning [16]. Previously, a survey of US postdoctoral periodontics programmes was conducted to assess whether CBCT was routinely incorporated into implant planning [17]. However, no studies are available on the use of CBCT in postgraduate periodontal diagnosis and treatment planning. Given the absence of formal guidelines on the role and implementation of CBCT in postgraduate periodontal education, there is an educational gap that needs to be addressed. Whilst keeping the ALARA principle and cost–benefit considerations in mind, this study can inform postgraduate periodontal education administrators on how the selective use of CBCT may influence infrabony defect topography assessment and Step 3 periodontal treatment planning in complex cases.

## 2. Materials and Methods

### 2.1. Ethical Clearance and Registration

The study protocol was approved by the Institutional Review Board of the University of Hong Kong/Hospital Authority Hong Kong West Cluster (IRB/REC Reference No. UW 23-549).

### 2.2. Participant Recruitment

#### 2.2.1. Study Population

Clinicians in periodontology from three different EFP-accredited centres were invited to participate in this study: King’s College London (KCL), United Kingdom; KU Leuven, Belgium; and The University of Hong Kong (HKU), Hong Kong SAR, China. First-year residents from all three centres were excluded from this study. The following clinicians were included: residents in second and third year; residents who have recently graduated from the postgraduate programme at HKU and are currently in the higher training stage of the specialist training pathway in Hong Kong; and specialists who hold, or have held, a position at one of the three centres.

#### 2.2.2. Sample Size Calculation

Based on the paired *t*-test, the sample size required to achieve 80% power to detect an effect size d = 0.5 (with a mean difference of 0.5 SD) at the 0.05 significance level was 34 participants. An effect size of 0.5 would indicate a moderate mean difference in scores for Questionnaires S1 and S2.

Based on the 2-sample *t*-test, at least 13 specialists and 25 residents were needed to achieve 80% power to detect an effect size d = 1 (with a mean difference of 1 SD) at the 0.05 significance level, assuming an allocation ratio of 1:2. An effect size of 1.0 would indicate a large mean difference in questionnaire scores between specialists and residents.

A study coordinator at each of the three participating centres was assigned and trained. By convenience sampling, each coordinator was responsible for inviting at least six residents in periodontology and six specialists in periodontology from their centre. Participants were invited to this study by invitation letter. The invitation included a study information sheet, which explained the nature of this study, and a written consent form. Participants gave consent by returning the completed written consent form to the study coordinators at the different centres.

### 2.3. Questionnaire Design

Questionnaires S1 and S2 for Participant 40 are included in the Supplementary Materials.

#### 2.3.1. Case Selection

Two questionnaires (identified as Questionnaires S1 and S2) were designed to fulfil the study aims and were distributed to participants in hard-copy format. Eight clinical cases (Case Numbers 1 to 8) were presented to the participants on two separate occasions. In Questionnaire S1, the cases were presented via conventional diagnostic records (intra-oral photographs, periodontal charting and periapical radiograph). In Questionnaire S2, CBCT images were included in addition to conventional data. Complex periodontal cases were selected from the pool of teaching patients, diagnosed with Stage III or IV periodontitis according to the 2017 World Workshop on the Classification of Periodontal and Peri-Implant Diseases and Conditions [18], under active care of residents in Periodontology at the Institute for Advanced Dentistry–Multi-Speciality Clinic, The University of Hong Kong (HKU IAD-MSC). By completing a written Teaching Patient’s Declaration Form, the teaching patients consented to and authorised HKU to use or share anonymised clinical data for research purposes with collaborating centres.

Patients were treated by residents in accordance with the EFP S3 level clinical practice guideline for treatment of Stage III or IV periodontitis [19,20]. Each of the eight clinical cases consisted of either a single-rooted tooth or a molar without furcation involvement and presented with residual deep probing pocket depth(s) of ≥6 mm associated with an infrabony defect after Step 1 and 2 therapy, requiring further treatment. The cases were only included if a full-mouth plaque score of ≤20% and full-mouth bleeding score of ≤25% were achieved after Step 1 and 2 therapy. Cases were excluded if (1) the teeth suffered from secondary occlusal trauma; (2) the location of the infrabony defects was at the third molars, distal surfaces of second molars, or at teeth with severe crowding or close root proximity; (3) the teeth presented with remarkable coronal or periapical features, such as developmental defects of enamel or hypercementosis; or (4) there were radiopaque coronal restorations or root canal obturation material at neighbouring teeth, resulting in CBCT artefacts. In all eight cases, a CBCT had already been prescribed by the resident-in-charge after Step 1 and 2 therapy for further treatment planning purposes or other reasons. All CBCT imaging was captured with a Planmeca ProMax 3D Mid imaging unit (Planmeca, Helsinki, Finland). The volume was acquired with the following standardised settings: a field of view of 10 cm × 10 cm; a voxel size of 0.2 mm; 120 kV; and 6.3 mA.

#### 2.3.2. Presentation of Case Data

Participants were provided with a clear straight 150 mm ruler of width 25 mm × height 160 mm, and a clear protractor of width 90 mm × height 45 mm (Raymay Fujii, Tokyo, Japan). For each case in Questionnaire S1, intra-oral photographs of the buccal and lingual or palatal view were provided. The periodontal charting included the following parameters: (1) gingival recession at six sites per tooth; (2) probing pocket depth (PPD) at six sites per tooth; (3) bleeding on probing (BoP) at six sites per tooth; (4) furcation involvement according to the Hamp et al. (1975) classification [21]; and (5) the degree of tooth mobility according to Miller’s Mobility Index (1950) [22]. A digital periapical radiograph was taken with a Morita Veraview iX intraoral X-ray system (Morita, Saitama, Japan) using a Dürr Image Plate Plus digital phosphor plate and imported into a Dürr VistaScan Mini view machine (Dürr Dental, Bietigheim-Bissingen, Germany). The image was exported in its original display settings and printed in its original size on the questionnaires for infrabony defect depth measurement. The exposure values used with the Morita Veraview iX short cone are listed in the Supplementary Materials as Supplementary Table S1. Periapical radiographs of posterior teeth were taken with size 2 image plates, whilst those of anterior teeth were taken with either size 0 or size 1 image plates, depending on the root morphology. In addition to the original radiograph of actual size, a copy of the radiograph was enlarged and printed in twice its original size for ease of viewing during infrabony defect angle measurement.

The same cases were presented again in Questionnaire S2. In addition to the conventional data, fixed CBCT images were also selected, saved, exported and printed in the original display settings from the Planmeca Romexis Viewer (Planmeca, Helsinki, Finland). For infrabony defect depth measurement, an axial view image of the infrabony defect was provided, containing a series of axial slices of a slice thickness of 0.2 mm and a slice separation of 0.5 mm. The number of axial slices included in the image depends on the infrabony defect depth. To ensure that the entire infrabony defect is captured in the image, the axial slices must extend apically beyond the base of the defect and coronally beyond the alveolar crest. In the same image, a coronal view of a slice thickness of 0.2 mm, aligned parallel to the long axis of the tooth of interest and bisecting the tooth mesiodistally, was provided for orientation; a 3D rendering image of the teeth and jaws was also provided for reference. For infrabony defect angle measurement, a sagittal view image of a slice thickness of 0.2 mm was provided, aligned parallel to the long axis of the tooth of interest. The sagittal slice capturing the base of the infrabony defect was selected as the sagittal view image.

#### 2.3.3. Defect Measurement Methods

**Figure 1:**
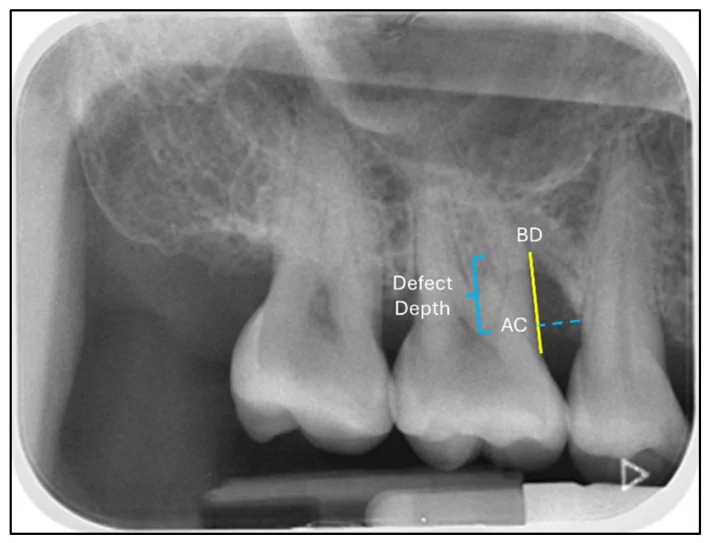
Infrabony defect depth equals the distance from point AC to point BD.

In Questionnaire S1, participants were instructed to measure the infrabony defect depth and angle on the periapical radiograph. For measuring infrabony defect depth, the method implemented by Li and coworkers was selected [23]. The defect depth equals the distance from the alveolar crest of the adjacent tooth (AC) to the base of the defect (BD), where AC is the intersection point across the root surface, when a perpendicular line is drawn from the alveolar crest of the adjacent tooth to the root surface of the involved tooth, and BD is the most apical extension of the infrabony defect where the periodontal ligament still retains its normal width [24]. A periapical radiograph of tooth 16 with a mesial infrabony defect was provided as an example measurement (Figure 1). This radiograph was obtained from a teaching case at the Periodontology Clinic at HKU IAD-MSC, which was not included as one of the eight cases in the questionnaires.

**Figure 2:**
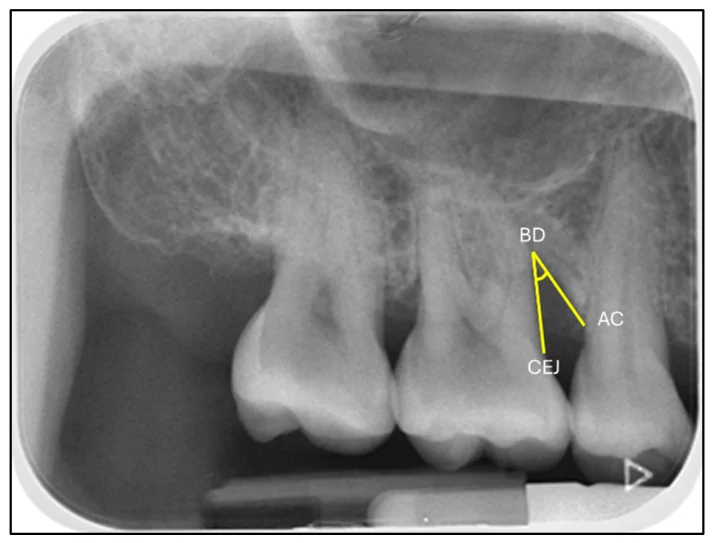
Infrabony defect angle equals the angle formed by the two lines AC–BD and BD–CEJ.

For measuring infrabony defect angle, the method implemented by Tonetti and coworkers was selected [25]. The angle of the infrabony defect equals the angle formed by the two lines AC–BD and BD–cementoenamel junction (CEJ) of the tooth of interest, where the line AC–BD represents the bone defect surface, and the line BD–CEJ represents the root surface of the involved tooth. The same periapical radiograph of tooth 16 was provided with an example measurement (Figure 2).

In Questionnaire S2, participants were instructed to measure the infrabony defect depth and angle of the same teeth of interest on the CBCT images.

The axial view image of the infrabony defect, containing a series of axial slices of a slice thickness of 0.2 mm and a slice separation of 0.5 mm, was provided to measure the defect depth. The formula for calculating the defect depth was provided:

Defect depth = (number of axial slices involving the defect − 1) × 0.5 mm + (0.2 mm/2) × 2

**Figure 3:**
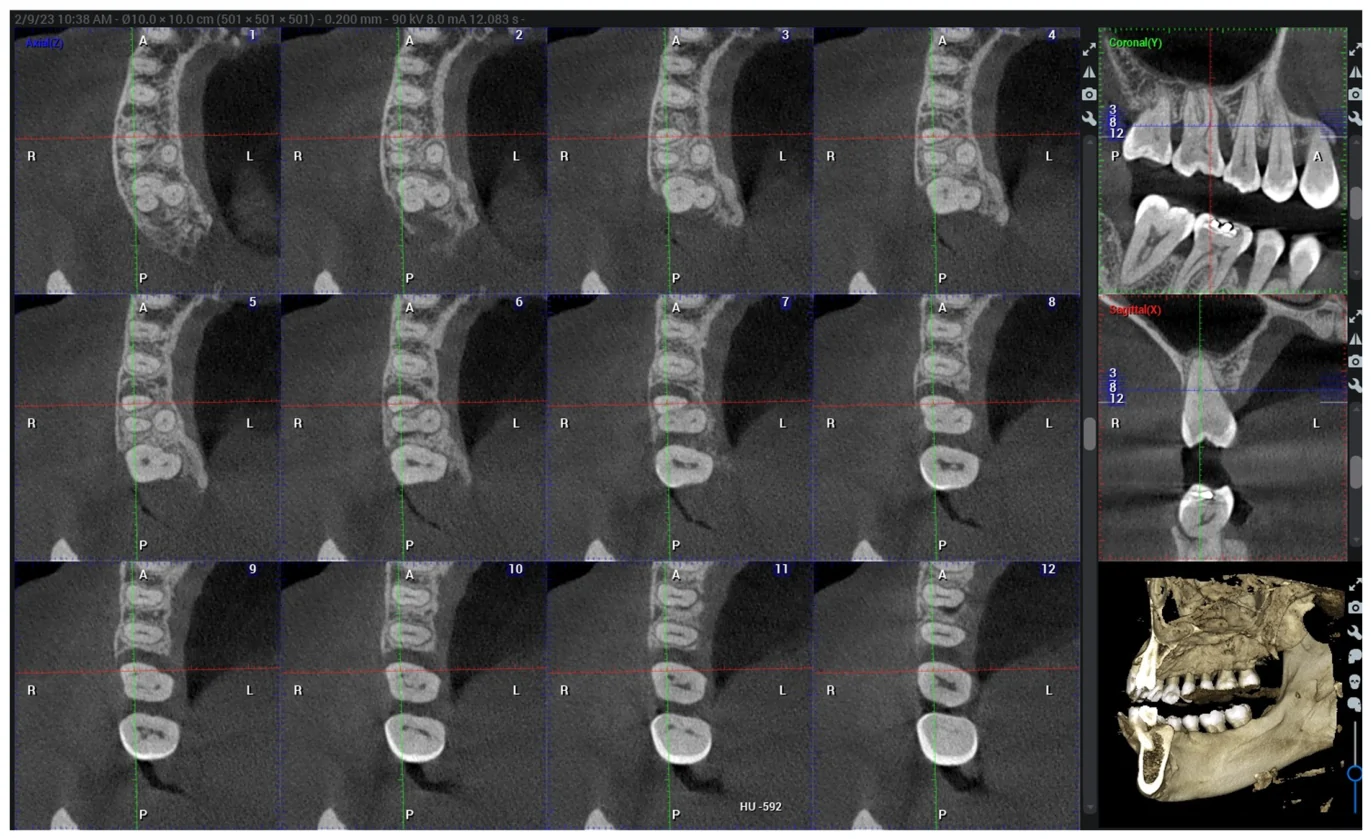
A CBCT axial view image of the infrabony defect at tooth 16 mesial.

Subtracting one from the number of axial slices capturing the infrabony defect will provide the number of slice separations involved. Each slice separation is 0.5 mm, which is then multiplied by the number of slice separations. Meanwhile, each slice thickness is 0.2 mm. The most coronal and apical axial slice capturing the defect is bisected by the slice separations in between. Therefore, 0.2 mm is divided by two to calculate the thickness of the most coronal and apical slice. The thickness, which is 0.1 mm, is then multiplied by two to include both slices in the total defect depth. Figure 3 displays the CBCT axial view image for the case example of tooth 16.

**Figure 4:**
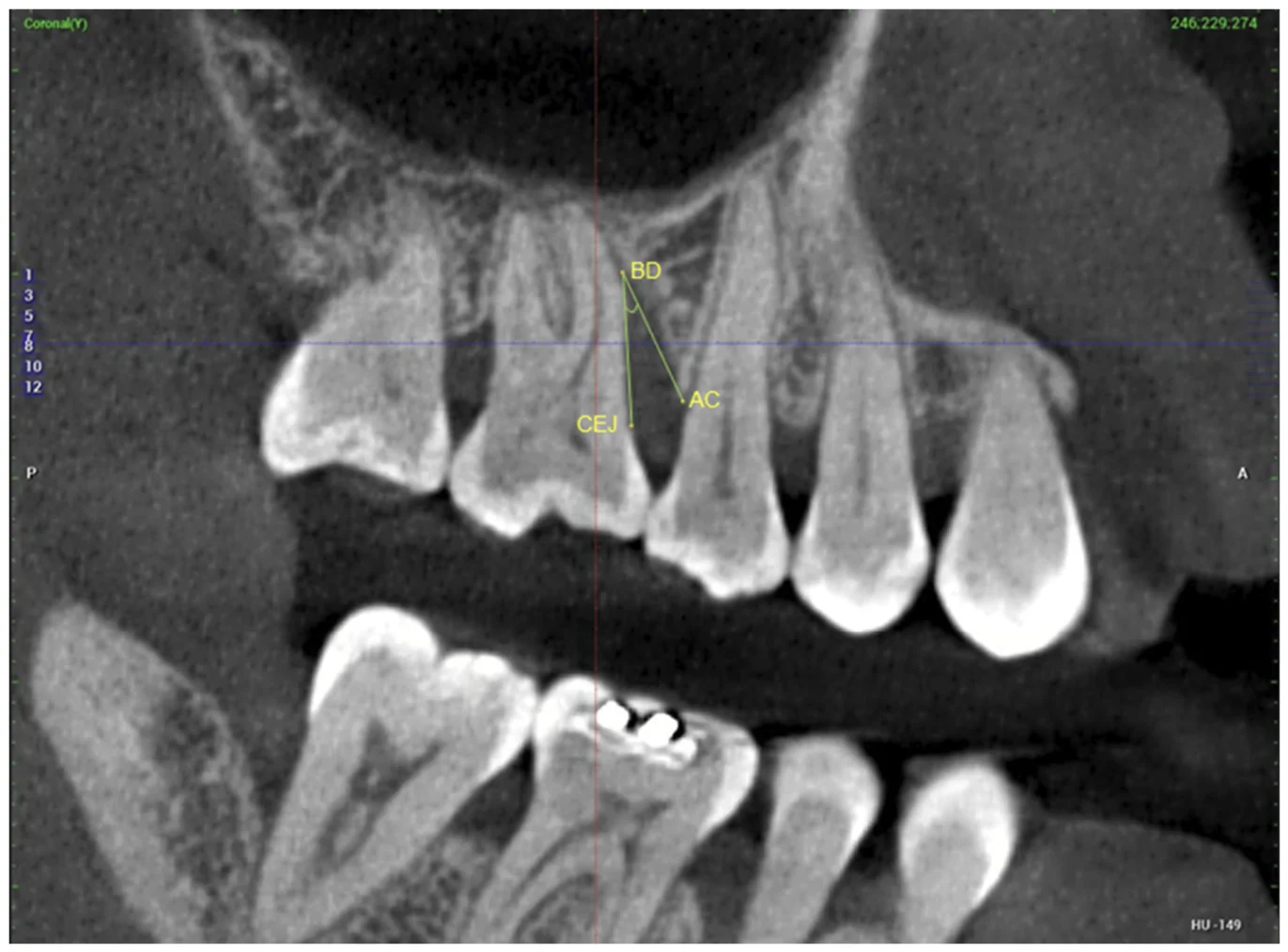
Infrabony defect angle measured on the CBCT sagittal view image. The defect angle equals the angle formed by the two lines AC–BD and BD–CEJ.

The sagittal view image of the infrabony defect was provided to measure the defect angle. The method for measuring the defect angle is identical to that instructed in Questionnaire S1. Figure 4 demonstrates defect angle measurement with the same case example of tooth 16.

#### 2.3.4. Formulation of Questions

For each case, the questions included in both Questionnaires S1 and S2 were identical. Question 1 recorded the participant’s level of experience (second-year resident; third-year resident; resident in higher training; or specialist). Questions 2 to 6 recorded participants’ assessment accuracy of the infrabony defect topography. Question 2 concerned defect extension around the root surface, whilst Question 3 concerned defect configuration. These two questions were designed in a multiple-choice format to allow for more nuanced data collection.

Question 4 concerned the defect depth. This question was designed in a dichotomous format. The participant was instructed to identify whether the defect depth was ≥3 mm (“Yes”/”No”). The demarcation was set at ≥3 mm based on current evidence: Nibali and coworkers recommended that periodontal regenerative surgery be considered the treatment of choice for deep residual pockets associated with infrabony defects ≥ 3 mm [26]. Therefore, based on this threshold, the participant’s measurement of the defect depth may subsequently influence their preferred management of the defect site.

Questions 5 and 6 concerned the defect angle. These two questions were also designed in a dichotomous format. The participant was instructed to identify whether the defect angle was ≤22 degrees (“Yes”/”No”) and ≥36 degrees (“Yes”/”No”). The demarcations set for the defect angle were based on the findings of Tsitoura and coworkers [27]. In periodontal regeneration surgery, with the use of enamel matrix derivatives in infrabony defects ≥3 mm, the probability of a clinical attachment level gain greater than 3 mm at one year post-operatively was significantly higher when the radiographic defect angle was ≤22 degrees compared with ≥36 degrees. It was, therefore, suggested that the baseline radiographic defect angle also be considered as a prognostic indicator of treatment outcome when opting for this regenerative approach. Therefore, Questions 5 and 6 were designed to distinguish participants’ accuracy in identifying the defect angle and assist participants in gauging the predictability of their treatment of choice, particularly if periodontal regeneration is considered.

Having completed the assessment of the defect topography, Questions 7 and 8 explored the participant’s preferred site management. These two questions were designed in multiple-choice format to capture the spectrum of clinician preferences in periodontal treatment planning.

#### 2.3.5. Scoring Format

A panel, consisting of two professorial staff in periodontology, supervised the periodontal surgical procedures for all eight cases included in the questionnaires. Both panel members were blinded to participants’ responses. The panel validated the following variables intra-surgically: defect extension around the root surface, defect configuration and defect depth. The panel derived the defect angle from CBCT assessment. The panel’s measurements were then used to provide reference-standard answers to Questions 2 to 6 in each case. As the questions for each case were identical in Questionnaires S1 and S2, the panel was invited to complete Questionnaire S2 only, as Questionnaire S2 contained all available case data (intra-oral photographs, clinical periodontal charting, periapical radiographs, and CBCT images). The panel was also permitted to simultaneously access Questionnaire S1 upon request. The panel finalised the reference-standard answers via a structured consensus process. When disagreements occurred, the two panel members reviewed the case together, referred to the predefined criteria, and resolved the disagreement through discussion, resulting in a reference-standard answer that reflected a joint decision.

A positive scoring system was adopted. No deduction of points was made for incorrect or blank answers. For each case, only Questions 2 to 6 were scored, as these questions focused on defect assessment and were of an objective nature. All participants’ answers to Questions 2 to 6 were, therefore, scored against the answers provided by the panel. Questions 7 and 8 focused on periodontal treatment planning concerning the tooth of interest; since treatment planning could be influenced by the participants’ treatment philosophy and clinical experience, these two questions were not scored.

In terms of the score breakdown, Question 2 was worth six points. If “Yes” was the correct answer to Q2a, the participant received one point by answering “Yes” and was then eligible to obtain a maximum of five more points in Q2b. In Q2b, the participant’s answers to each of the five options (Q2b.1–5) were scored, with correct discernment of each option being worth one point each. If “No” was the correct answer to Q2a, the participant received six points directly for answering “No”. In this scenario, if the participant incorrectly answered “Yes” to Q2a, the participant would not obtain any points for Question 2. Similarly, Question 3 was worth five points. Correct discernment of each of the five options (Q3.1–5) was worth one point each. Questions 4 to 6 were worth one point each. Therefore, a maximum of fourteen points could be obtained for each case. This case score was then expressed as a percentage score. As there were eight cases in each questionnaire, the participant’s questionnaire score was, therefore, the mean of the eight percentage scores.

### 2.4. Study Procedure

At each centre, a study coordinator (a department staff member at each respective centre who was not one of the study authors) was assigned, who then allocated each participant an individual participant number. This ensured the anonymity of the participants to the study investigator.

The order of completion of Questionnaires S1 and S2, as well as the order of the eight cases within each questionnaire, were randomised for each participant using Microsoft Excel (Microsoft, Redmond, WA, USA). The study investigator then printed and compiled the randomised order for each participant into two binders (one binder for Questionnaire S1 and one binder for Questionnaire S2). The binders and stationery for the HKU participants were delivered in person by the HKU study coordinator. The binders and stationery for the overseas participants were delivered from Hong Kong to KCL and KU Leuven via international registered mail. After the participants completed their first questionnaires, a washout period of 2 weeks was then enforced by the study coordinators. The second questionnaires were completed by the participants after the washout period. The study coordinators were trained to provide support to the participants throughout the entire process.

Participants were instructed to complete each questionnaire within one week, at their own convenience. Participants were instructed not to leave any questions unanswered. For each case, participants were instructed to write down the start time and end time, in hours and minutes. This information was then processed by the study investigator to calculate the time taken by the participant to complete each case.

At HKU, the completed questionnaires were returned in person to the study coordinator, who then delivered the questionnaires in person to the study investigator. At KCL and KU Leuven, the completed questionnaires were returned in person to the study coordinator, who then scanned the questionnaires and transferred the digital copies to the study investigator via a secure cloud storage service. All data were then processed and analysed by the study investigator.

### 2.5. Statistical Methods

All data were extracted from the questionnaires and processed using Microsoft Excel. Missing answers in the questionnaires were excluded from statistical analysis. The sample size was calculated with the statistical software G*power 3.1.9.7 [28]. The statistical analysis was performed using IBM SPSS Statistics Version 27 (IBM, New York, NY, USA).

The Shapiro–Wilk test was performed to determine if the continuous variables were normally distributed. The time taken for Questionnaire S1 did not follow a normal distribution. Levene’s test was performed to assess the equality of variances across groups. There was unequal variance across resident and specialist groups for the time taken to complete Questionnaire S2.

To compare the participants’ infrabony defect topography assessment accuracy with conventional periodontal diagnostics versus with CBCT, the paired *t*-test was selected to compare the mean scores for Questionnaires S1 and S2 at a significance level of 0.05. To compare accuracies in defect assessment between residents and specialists, with and without CBCT, the 2-sample *t*-test was selected to compare the mean difference between the two groups for both Questionnaire S1 and S2 scores, at a significance level of 0.05. To identify whether one of the groups benefited more significantly from CBCT, the 2-sample *t*-test was selected to compare the mean difference in score differentials (Questionnaires S1 and S2) between the two groups, at a significance level of 0.05.

To compare the time taken for infrabony defect assessment and treatment planning, once with conventional periodontal diagnostics, and once with CBCT as an adjunct, the Wilcoxon signed-rank test was selected to compare the time taken for Questionnaires S1 and S2 at a significance level of 0.05. To compare the time taken for infrabony defect assessment between residents and specialists, with and without CBCT, the 2-sample *t*-test was selected to compare the time taken for Questionnaires S1 and S2 and the differential in time taken (Questionnaires S1 and S2) between the two groups, at a significance level of 0.05.

To compare participants’ decisions in Step 3 periodontal treatment planning, once with conventional periodontal diagnostics and once with the addition of CBCT, a measure of agreement was calculated by case. Cohen’s kappa value was calculated for each case to identify whether incorporating CBCT into defect assessment altered participants’ treatment of choice for the defect site. Furthermore, for each case, if periodontal regeneration was retained as the treatment of choice in both questionnaires, a kappa value was calculated for the choice of flap design and biomaterials to investigate agreement on periodontal regeneration strategy.

## 3. Results

### 3.1. Participant Profile

Forty out of 46 participants returned the questionnaires. The response rate was, therefore, 87.0%. Twenty-five participants were residents in periodontology (14 were second-year residents, six were third-year residents, and five were residents in higher training). Fifteen participants were specialists in periodontology. Table 1 displays the participant profile, with distribution by status and centre.

### 3.2. Questionnaire Scores

The mean score (%) was 63.5 ± 11.8 for Questionnaire S1 and 69.8 ± 11.9 for Questionnaire S2. The mean difference in scores (%) was −6.3 ± 8.0, which was statistically significant (*p* < 0.001). Therefore, the use of CBCT for infrabony defect topography assessment yields a statistically significant improvement in diagnostic accuracy when compared with conventional periodontal diagnostics alone. However, the clinical relevance of this magnitude of improvement cannot be determined from the present study. Establishing the clinical significance of adjunctive CBCT assessment will require a study design that includes analysis of case treatment outcomes based on clinicians’ defect assessment, clinical decision making and treatment planning, in cases evaluated with and without pre-operative CBCT. Table 2 displays the results.

In this study, no statistically significant differences were found when comparing mean questionnaire scores between residents and specialists. For Questionnaire S1, the mean score (%) was 63.7 ± 12.0 for residents and 63.2 ± 12.1 for specialists. The mean difference in scores (%) was 0.5 (95% CI: −7.4–8.5), which was not statistically significant (*p* > 0.05). For Questionnaire S2, the mean score (%) was 68.9 ± 12.9 for residents and 71.4 ± 10.2 for specialists. The mean difference in scores (%) was −2.5 (95% CI: −10.4–5.5), which was not statistically significant (*p* > 0.05). Residents and specialists were also compared for the mean score differential between Questionnaires S1 and S2. The mean score differential (%) in questionnaires was −5.2 ± 7.9 for residents and −8.2 ± 8.2 for specialists. The mean difference in score differentials (%) between the two groups was 3.0 (95% CI: −2.3–8.3), which was not statistically significant (*p* > 0.05). This suggests that the extent of additional benefit provided by CBCT in infrabony defect topography assessment was similar for both resident and specialist groups. However, the limited sample size reduces the ability to detect statistically significant between-group differences, and larger studies are needed to validate these results. Table 3 displays the results.

### 3.3. Time Taken to Complete the Questionnaires

There was no statistically significant difference in the median time taken for participants to complete Questionnaires S1 and S2. The mean time taken was 30.8 ± 14.5 min for Questionnaire S1 and 35.3 ± 13.5 min for Questionnaire S2. The median time taken was 27.5 min for Questionnaire S1 and 32.5 min for Questionnaire S2. The difference in median time taken was −3.5 min (*p* > 0.05). Table 4 displays the results.

The mean time taken to complete Questionnaire S1 was 33.8 ± 15.5 min for residents and 25.9 ± 11.5 min for specialists. The mean difference in time taken was 7.9 min (95% CI: −1.5–17.2), which was not statistically significant (*p* > 0.05). For Questionnaire S2, the mean time taken was 33.8 ± 10.7 min by residents and 38.0 ± 17.3 min by specialists. The mean difference in time taken was −4.2 min (95% CI: −14.6–6.0), which was not statistically significant (*p* > 0.05).

There was a statistically significant difference when comparing residents and specialists for their respective differential in time taken between Questionnaires S1 and S2. The differential in time taken between questionnaires was 0.0 ± 13.9 min for residents and −12.1 ± 12.9 min for specialists. The mean difference in differential time taken between groups was 12.1 min (95% CI: 3.2–21.1), which was statistically significant (*p* < 0.05). This difference suggests that compared with specialists, residents spent significantly less additional time interpreting CBCT images alongside conventional periodontal diagnostic records during infrabony defect assessment. Table 5 displays the results.

### 3.4. Periodontal Treatment Planning

In all eight cases, there was a proportion of participants whose treatment of choice for the same defect differed when provided with conventional periodontal diagnostic records (Questionnaire S1), and when provided with CBCT images in addition to conventional diagnostics (Questionnaire S2). The proportion of participants who altered their treatment of choice ranged from 15.0% (Case 5) to 50.0% (Case 1). Table 6 displays the results.

Within the participants who retained periodontal regeneration as their treatment of choice for the same case on both questionnaires, there was a proportion who altered their choice of flap design or biomaterials. Missing data were present in this subgroup and were excluded from the analysis. In Case 5, one participant did not provide an answer for the choice of flap design, whilst one participant did not provide an answer for the choice of biomaterials. The same was observed for Case 7. Therefore, two participants in Case 5 and two participants in Case 7 were excluded from the subgroup analysis. Case 1 was excluded as only one participant was eligible for further analysis, which was insufficient for calculating proportions. Therefore, the proportion of participants who altered their choice of flap design ranged from 15.4% (Case 2) to 73.3% (Case 7). Likewise, the proportion of participants who altered their choice of biomaterials ranged from 14.3% (Case 8) to 50% (Case 6). Table 7 displays the results.

The measure of agreement between conventional periodontal diagnostics (Questionnaire S1) and CBCT (Questionnaire S2) on periodontal treatment planning was investigated by case, as displayed in Table 8. Cohen’s kappa value for treatment of choice ranged from 0.001 (Case 1) to 0.472 (Case 6). Therefore, the degree of agreement between conventional periodontal diagnostics and CBCT on treatment of choice ranged from no agreement to moderate agreement. For participants who retained periodontal regeneration as their treatment of choice for the same case in both questionnaires, the measures of agreement on choice of flap design and biomaterials were investigated. Cases 1 (*n* = 1) and 6 (*n* = 2) were excluded due to insufficient participants available for the measure of agreement. For choice of periodontal regeneration flap design, the kappa value ranged from −0.111 (Case 2) to 0.575 (Case 4). The degree of agreement between conventional periodontal diagnostics and CBCT on the choice of regenerative flap design, therefore, ranged from no agreement to moderate agreement. For choice of biomaterials, the kappa value ranged from −0.083 (Case 3) to 0.652 (Case 5). The degree of agreement between conventional periodontal diagnostics and CBCT on the choice of biomaterials also varied widely, ranging from no agreement to substantial agreement.

The wide range of degree of agreement suggests that in cases requiring further treatment after Step 1 and 2 therapy, adjunctive CBCT assessment of the infrabony defect topography may lead to changes in treatment approach compared with when deciding with conventional diagnostics alone. Furthermore, CBCT assessment of the infrabony defect topography may lead to changes in the choice of flap design and biomaterials when periodontal regeneration is considered for site management.

## 4. Discussion

With judicious case selection and adherence to the ALARA principle, CBCT used as an adjunct to conventional periodontal diagnostics provided a statistically significant improvement in assessing infrabony defect topography. However, whether this translates into any clinically significant improvement remains unknown. Establishing clinical significance will require a larger-scale study incorporating analysis of clinical and radiographic treatment outcomes based on clinicians’ defect assessments and treatment decisions, with and without the use of CBCT.

CBCT has an extensive evidence base beyond clinical periodontology, with its diagnostic and treatment-planning utility widely demonstrated in other fields such as dental implantology, endodontics, and oral and maxillofacial surgery. In the 2019 European Society of Endodontology position statement, ‘Use of Cone-Beam Computed Tomography in Endodontics’, the authors concluded that, when applied on a case-by-case basis and in accordance with the ALARA principle, CBCT can support diagnosis, aid treatment planning, and enhance clinical management across a range of endodontic scenarios [29]. The authors further recommended that dental undergraduate and endodontic postgraduate programmes incorporate CBCT-related education into their curricula, including training in justification, operation, interpretation, and reporting of CBCT imaging [29]. In the present study, adjunctive CBCT use demonstrated greater diagnostic accuracy in assessing periodontal infrabony defect sites and may lead to changes in site management compared with conventional periodontal diagnostics alone, particularly in relation to flap design and biomaterial selection when periodontal regeneration is considered. Consistent with the 2019 European Society of Endodontology position statement, these findings highlight the importance of defining the role of CBCT within postgraduate periodontal education, especially with respect to appropriate case selection and the promotion of judicious, evidence-based use of CBCT in the diagnosis and management of complex periodontal cases.

In this present study, optimal quality of the CBCT images may have helped enhance the clinician’s assessment ability. Acquisition parameters such as milliamperage, kilovoltage and voxel size may influence the quality of the CBCT images. Voxel size is a key factor in determining the spatial resolution of an image. Smaller voxel sizes improve image resolution and detail, but at the cost of an increased radiation dose. The principle of ALARA requires that clinicians maintain ionising radiation as low as reasonably achievable for patients. Therefore, both diagnostic accuracy and radiation dose should be well considered when prescribing CBCT. Dong and coworkers reported that whilst there was no statistically significant difference in diagnostic value between 0.125 mm and 0.2 mm voxel sizes for detecting alveolar bone defects, a 0.4 mm voxel size was of a significantly lower diagnostic value compared with both 0.125 mm and 0.2 mm voxel sizes respectively [30]. The authors, therefore, proposed that a 0.2 mm voxel size was an appropriate setting for the detection of alveolar bone defects with CBCT. Only CBCT volumes acquired with a 0.2 mm voxel size were retrieved for this study. The diagnostic clarity offered by CBCT images of a 0.2 mm voxel size may contribute to increased accuracy in assessing periodontal infrabony defect topography when compared with conventional periodontal diagnostics alone, which was a significant finding in this study.

Despite using optimal CBCT acquisition parameters, a limitation of the questionnaire design was that both the CBCT and periapical images were printed and assessed on paper rather than viewed digitally. This constraint may have influenced the differentiation between residents and specialists in their infrabony defect assessment accuracy. In routine clinical practice, digital viewers allow clinicians to enlarge images, adjust contrast and brightness, and navigate through CBCT slices to better appreciate the region of interest. Restricting participants to a static, non-adjustable printed format may have limited their ability to utilise these tools for fine-detail interpretation. Therefore, the use of printed images may have attenuated any performance gap between the two groups and should be considered when interpreting the comparative accuracy results. Nevertheless, standardisation challenges across multiple centres would be anticipated if the diagnostic images were presented on a digital viewer. Challenges would include standardising the electronic device, model, screen size, display settings (involving display aspect ratio, resolution, brightness, and colour profiles) and ambient lighting conditions for participants across all centres when they view the diagnostic images. These factors can introduce uncontrolled variability between centres. To minimise these sources of heterogeneity, this study adopted printed CBCT and periapical radiograph images together with standardised stationery for measurements. Although this approach does not replicate the full functionality of digital viewers, it ensured that all participants across the three centres assessed the diagnostic data under identical, standardised conditions. This inter-centre standardisation was essential for the comparative design of this study.

Another limitation of this study was the use of convenience sampling, which was adopted due to the accessibility and availability of clinicians with different levels of experience within the institutional setting. This approach introduces potential sampling bias, particularly among specialists, whose treatment philosophies may vary according to clinical experience and training background. To mitigate this limitation, clinicians of varying experience were recruited across three centres to enhance the generalisability of the findings. Future larger-scale studies incorporating simple random sampling of clinicians appointed at each centre within a defined time period would further reduce sampling bias and strengthen the robustness of the conclusions.

In the questionnaires, each clinical case represents a unique and complex infrabony defect scenario with distinct tooth-level and site-level characteristics. Consequently, agreement between conventional periodontal diagnostics (Questionnaire S1) and CBCT (Questionnaire S2) for periodontal treatment planning was not assessed using a pooled Cohen’s kappa analysis, given heterogeneity in defect morphology and site-specific characteristics across cases. Case-specific kappa values are reported instead, as they are more directly interpretable for individual clinical scenarios.

This study reveals that the treatment of choice for the same infrabony defect may change after CBCT is utilised as an adjunctive diagnostic modality. Periodontal regeneration strategies, involving various combinations of flap designs and biomaterials, may change after assessing the infrabony defect topography on the CBCT. For example, the clinical periodontal charting and CBCT images can be interpreted together to confirm whether the infrabony defect extension is strictly limited to one to two surfaces of the root, which then prompts the selection of an M-MIST or MIST flap design, depending on whether sufficient access from the buccal aspect is anticipated [31,32]. The choice of biomaterials for periodontal regeneration also depends on the infrabony defect topography. For example, Cortellini and Tonetti recommended that enamel matrix derivatives (EMDs) can be used alone in defects with a prevalent three-wall morphology or in well-supported two-wall defects [33]. In non-containing defects, a combination of EMDs and a filler, with or without a barrier membrane, is generally recommended. As CBCT can provide intricate information on infrabony defect configuration and extension, it can assist the clinician in determining the most suitable flap design and biomaterials for periodontal regeneration. This conclusion is in alignment with a recent narrative review suggesting that CBCT may aid in determining the applicability of periodontal regenerative therapy and predicting surgical outcomes [1]. Nevertheless, this study evaluated diagnostic correctness, and it was not reported whether CBCT utility leads to improved clinical decisions or superior post-operative clinical outcomes. The cost–benefit of integrating CBCT in pre-operative assessment, as well as the magnitude of improvement in clinical outcomes, can be investigated in further studies with an improved study design.

To explore the applicability of the current findings to other types of periodontal osseous defects, such as in cases of interradicular bone loss, future research could be conducted with an extended case variety. Currently, there is evidence demonstrating a high accuracy of CBCT in classifying the degree of furcation involvement in maxillary molars [34]. A pilot clinical study also concluded that CBCT assessments of maxillary teeth with furcation involvement resulted in reduced costs and treatment times in Switzerland [35]. In terms of the vertical depth of furcation, a recent study revealed that measurements with conventional periodontal diagnostics based on periapical radiographs and clinical periodontal chartings resulted in poor to fair agreement with CBCT [36]. It would, therefore, be of interest to include cases of both maxillary and mandibular molars associated with furcation involvement and different degrees of vertical furcation subclassification, presented with residual deep probing pocket depth(s) of ≥6 mm after Step 1 and 2 therapy, in future multi-centre studies of a larger scale to consolidate such evidence.

## 5. Conclusions

Within the limitations of the present study, the following conclusions were drawn:

1. With judicious case selection and adherence to the ALARA principle, CBCT used as an adjunct to conventional periodontal diagnostics provided a statistically significant improvement in assessing periodontal infrabony defect topography. However, whether the magnitude of this improvement is clinically meaningful requires further investigation. These findings reinforce that CBCT should remain a selective adjunct rather than a routine replacement for conventional two-dimensional radiographic assessment.
2. Although there was no statistically significant difference in the assessment accuracy of infrabony defect topography, with and without CBCT, between residents and specialists in Periodontology, this study was underpowered to detect statistically significant between-group differences, and larger studies are needed before reaching definitive conclusions.
3. After completion of Step 1 and 2 therapy, adjunctive CBCT assessment of the defect site may lead to changes in site management when compared with assessment with conventional periodontal diagnostics alone.
4. This study highlights several areas requiring further investigation. Although the adjunctive use of CBCT demonstrated greater diagnostic accuracy in assessing infrabony defect sites, additional research is needed to determine whether this translates into superior clinical outcomes or improved tooth prognosis. The findings also underscore the importance of clarifying the role of CBCT within postgraduate periodontal education, particularly in relation to appropriate case selection and the promotion of judicious, evidence-based use of CBCT in diagnosis and management of complex periodontal cases. A larger-scale study with an enhanced methodological design and the inclusion of additional EFP-accredited centres would enable more robust and generalisable conclusions.

## Figures and Tables

**Table 1:** Participant profile.

Participants					
Status		Centre			Total *n* = 40 (100%)
		HKU *n* = 20 (50%)	KCL *n* = 12 (30%)	KU Leuven *n* = 8 (20%)	
Resident	Second Year	5 (25%)	6 (50%)	3 (37.5%)	14 (35%)
	Third Year	3 (15%)	0 (0%)	3 (37.5%)	6 (15%)
	Higher Training	5 (25%)	N/A	N/A	5 (12.5%)
Specialist		7 (35%)	6 (50%)	2 (25%)	15 (37.5%)

**Table 2:** Questionnaires S1 and S2 scores (%).

	Mean ± Standard Deviation	95% Confidence Interval		Minimum	Maximum	Range	*p*-Value ^†^
		Lower	Upper				
Questionnaire S1	63.5 ± 11.8	59.7	67.3	41.1	86.6	45.5	<0.001 ***
Questionnaire S2	69.8 ± 11.9	66.0	73.7	44.6	89.3	44.6	
Difference (Q1–Q2)	−6.3 ± 8.0	−8.9	−3.7	−22.3	11.6	33.9	

^†^: *p*-value is obtained from the paired *t*-test. *** *p* < 0.001.

**Table 3:** Resident vs. specialist scores (%).

	Resident Mean ± Standard Deviation	Specialist Mean ± Standard Deviation	Mean Difference	95% Confidence Interval of the Difference		*p*-Value ^†^	Effect Size (d)	Post Hoc Power
				Lower	Upper			
Questionnaire S1	63.7 ± 12.0	63.2 ± 12.1	0.5	−7.4	8.5	0.892	0.04	0.05
Questionnaire S2	68.9 ± 12.9	71.4 ± 10.2	−2.5	−10.4	5.5	0.534	0.21	0.10
Difference (Q1–Q2)	−5.2 ± 7.9	−8.2 ± 8.2	3.0	−2.3	8.3	0.261	0.37	0.20

^†^: *p*-values were obtained from the 2-sample *t*-test.

**Table 4:** Time taken for Questionnaires S1 and S2 (min).

	Mean ± Standard Deviation	95% Confidence Interval		Median	Minimum	Maximum	Range	*p*-Value ^†^
		Lower	Upper					
Questionnaire S1	30.8 ± 14.5	26.1	35.5	27.5	9.0	69.0	60.0	
Questionnaire S2	35.3 ± 13.5	31.0	39.7	32.5	14.0	78.0	64.0	
Difference (Q1–Q2)	−4.5 ± 14.6	−9.2	0.1	−3.5	−40.0	28.0	68.0	0.082

^†^: *p*-value was obtained from the Wilcoxon signed ranks test.

**Table 5:** Time taken for resident vs. specialist (min).

	Resident Mean ± Standard Deviation	Specialist Mean ± Standard Deviation	Mean Difference	95% Confidence Interval of the Difference		*p*-Value ^†^	Effect Size (d)	Post Hoc Power
				Lower	Upper			
Questionnaire S1	33.8 ± 15.5	25.9 ± 11.5	7.9	−1.5	17.2	0.099	0.58	0.41
Questionnaire S2	33.8 ± 10.7	38.0 ± 17.3	−4.2	−14.6	6.0	0.397	0.29	0.14
Difference (Q1–Q2)	0.0 ± 13.9	−12.1 ± 12.9	12.1	3.2	21.1	0.009 **	0.90	0.77

^†^: *p*-values were obtained from the 2-sample *t*-test. ** *p* < 0.01.

**Table 6:** Periodontal treatment planning after Step 1 and 2 therapy.

Case No.	N	Treatment of Choice for Questionnaires S1 and S2	
		Choice Retained (*n*/%)	Choice Altered (*n*/%)
1	40	20 (50.0%)	20 (50.0%)
2	40	23 (57.5%)	17 (42.5%)
3	40	26 (65.0%)	14 (35.0%)
4	40	23 (57.5%)	17 (42.5%)
5	40	34 (85.0%)	6 (15.0%)
6	40	26 (65%)	14 (35.0%)
7	40	24 (60%)	16 (40%)
8	40	31 (77.5%)	9 (22.5%)

**Table 7:** Periodontal regeneration treatment planning after Step 1 and 2 therapy.

Case No.	If Periodontal Regeneration was Retained as the Treatment of Choice for Both Questionnaires:				
	N	Choice of Flap Design		Choice of Biomaterials	
		Retained (*n*/%)	Altered (*n*/%)	Retained (*n*/%)	Altered (*n*/%)
1	1	0 (0.0%)	1 (100.0%)	1 (100.0%)	0 (0.0%)
2	10	7 (70.0%)	3 (30.0%)	8 (80.0%)	2 (20.0%)
3	13	11 (84.6%)	2 (15.4%)	10 (76.9%)	3 (23.1%)
4	19	15 (78.9%)	4 (21.1%)	15 (78.9%)	4 (21.1%)
5	33	20 (60.6%)	13 (39.4%)	28 (84.8%)	5 (15.2%)
6	2	1 (50.0%)	1 (50.0%)	1 (50.0%)	1 (50.0%)
7	15	4 (26.7%)	11 (73.3%)	12 (80%)	3 (20%)
8	28	20 (71.4%)	8 (28.6%)	24 (85.7%)	4 (14.3%)

**Table 8:** Measurement of agreement in periodontal treatment planning.

Case No.	Treatment of Choice in Questionnaires S1 and S2	If Periodontal Regeneration was Retained as the Treatment of Choice in Both Questionnaires:	
		Choice of Flap Design	Choice of Biomaterials
	Cohen’s Kappa Value (95% CI)		
1	0.001 (−0.136, 0.137)	N/A	
2	0.418 (0.214, 0.621)	−0.111 (−0.274, 0.052)	−0.154 (−0.374, 0.066)
3	0.451 (0.238, 0.664)	0.435 (−0.189, 1.059)	−0.083 (−0.206, 0.039)
4	0.257 (−0.027, 0.542)	0.575 (0.265, 0.886)	0.550 (0.195, 0.905)
5	0.329 (−0.055, 0.713)	0.268 (−0.025, 0.561)	0.652 (0.398, 0.906)
6	0.472 (0.269, 0.674)	N/A	
7	0.243 (−0.017, 0.504)	0.478 (0.319, 0.636)	0.575 (0.203, 0.948)
8	0.523 (0.279, 0.766)	0.596 (0.373, 0.820)	0.299 (0.091, 0.507)

## Data Availability

The anonymised dataset underlying the analyses is available from the corresponding author upon reasonable request.

## Supplementary Materials

The following supporting information can be downloaded at https://www.mdpi.com/article/10.3390/dj14090576/s1: File S1: Questionnaire S1; Questionnaire S2; and Table S1: Intra-oral radiograph exposure values used with short cone.
