# Supplementary material for: Does Cone-Beam Computed Tomography Provide Additional Benefit to Periodontal Infrabony Defect Topography Assessment? A Multi-Centre Case-Based Questionnaire
Source: Dent J (Basel). 2026 Sep 8;14(9):576. doi: 10.3390/dj14090576 (PMC13605316; doi:10.3390/dj14090576)
Supplement: Supplementary file 1 [file dentistry-14-00576-s001.zip › File S1.pdf]

Supplementary Information

Participant Number: 40

Questionnaire S1

Questionnaire S1 contains 8 cases with completion of Step 1 & 2 therapy. In each case, the location of the periodontal defect is specified. The following information is given for each case:

- *Periodontal re-evaluation clinical and radiographic data:*
  - *Periodontal charting*
    - *Parameters:*
      - *6-point PPD, recession, BOP*
      - *Furcation involvement (Hamp et al.)*
      - *Tooth hypermobility (Miller)*
      - *Presence of fremitus*
  - *Periapical radiograph(s) (one is scaled to actual size)*
  - *Intra-oral photographs*

For measuring the depth of the infrabony defect, please use the following method (Li, Jia & Ouyang 2015):

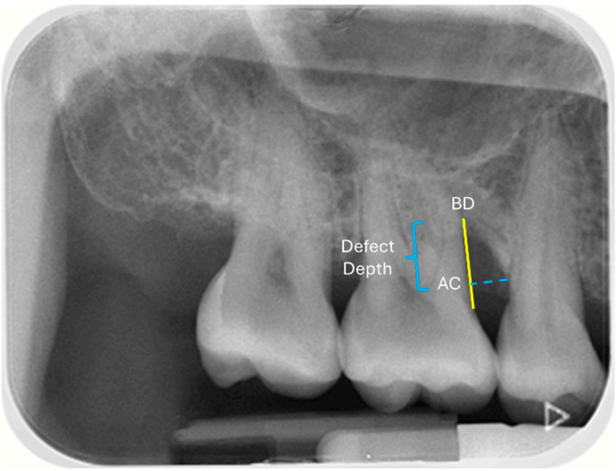

*Defect depth = distance from point **AC** to point **BD**, where:*

- ***AC** (**A**lveolar **C**rest of adjacent tooth) is the intersection point across the root surface, when a perpendicular line is drawn from the alveolar crest of the adjacent tooth to the root surface of the involved tooth*
- ***BD** (base of the defect) is the most apical extension of the infrabony defect where the PDL still retains its normal width (Bjorn et al. 1969)*

For measuring the angle of the infrabony defect, please use the following method (Tonetti et al. 1993):

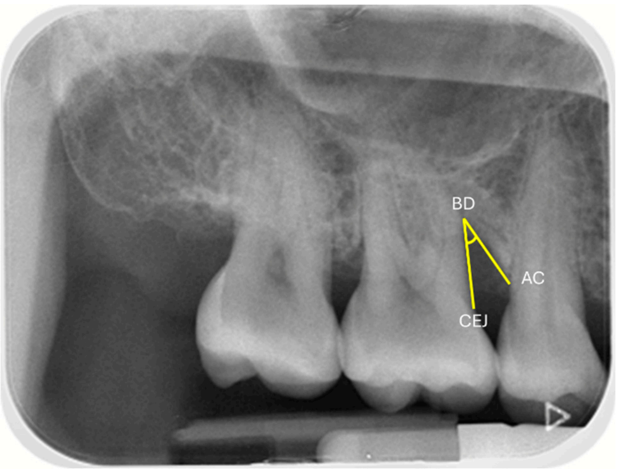

Angle of defect = the angle formed by the two lines ***AC*-*BD*** *and* ***BD*-*CEJ****, where:*

- ***BD*-*CEJ*** *= the root surface of the involved tooth*
- ***AC*-*BD*** *= the bone defect surface*

Finally, please kindly complete all the cases and **note down the start and end time** *for each case.*

Thank you for your participation.

Start time: ________________

16 mesial

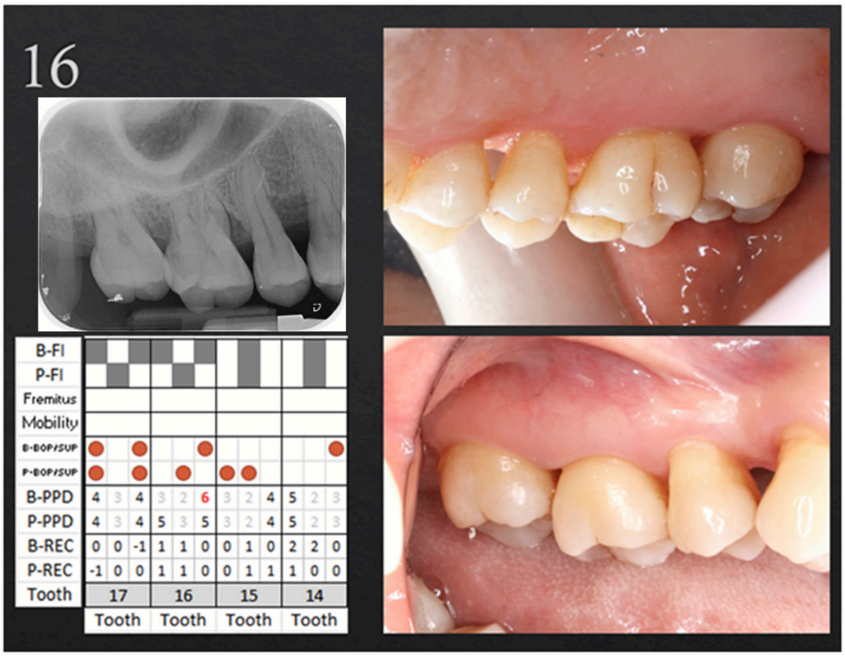

B-FI												
P-FI												
Fremitus												
Mobility												
B-BOP/SUP												
P-BOP/SUP												
B-PPD	4	3	4	3	2	6	3	2	4	5	2	3
P-PPD	4	3	4	5	3	5	3	2	4	5	2	3
B-REC	0	0	-1	1	1	0	0	1	0	2	2	0
P-REC	-1	0	0	1	1	0	0	1	1	1	0	0
Tooth	17			16			15			14		
	Tooth			Tooth			Tooth			Tooth		

The periapical radiograph above is scaled to actual size. Please perform your depth measurement on this radiograph.

The periapical radiograph below is magnified for better viewing. You may perform your angle measurement on this radiograph.

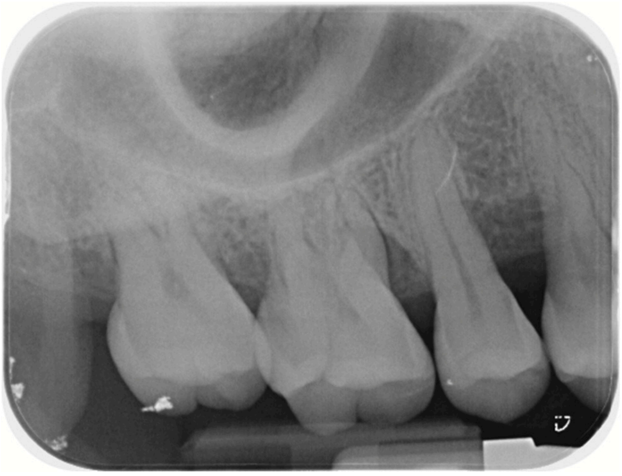

Please enter the start time for this case questionnaire at the top left corner.

1. **Are you currently a:**
   ☐ Second-year resident
   ☐ Third-year resident
   ☐ Resident in higher training
   ☐ Specialist

2. **Does the defect involve:**
   a. More than 1 surface of the root of the same tooth? ☐ Yes ☐ No
   [If you answered "No" for a), please proceed to Q3]
   b. If you answered "Yes" for a), which surfaces of the root does the defect involve? You may choose more than one answer:
   ☐ Mesial and buccal
   ☐ Mesial and palatal
   ☐ Mesial - buccal - distal
   ☐ Mesial - palatal - distal
   ☐ Circumferential

3. **Is there a presence of the following type(s) of infrabony defect? You may choose more than one answer:**
   ☐ 1-walled component
   ☐ 2-walled component
   ☐ 3-walled component
   ☐ Crater
   ☐ Circumferential defect

4. **Is the depth of the defect ≥ 3mm?**
   ☐ Yes ☐ No

5. **Is the angle of the defect ≤ 22 degrees?**
   ☐ Yes ☐ No

6. **Is the angle of the defect ≥ 36 degrees?**
   ☐ Yes ☐ No

7. **Simply considering the defect presentation alone, which procedure do you think is the most appropriate for site management? Please choose only one option:**
   ☐ Non-surgical maintenance
   ☐ Access flap
   ☐ Access flap with osseous resective surgery
   ☐ Periodontal regeneration
   ☐ Tooth extraction
   ☐ Others (Please specify: ____)

8. If periodontal regeneration is selected for Question 7:

a. Which regenerative approach would you select? Please choose only one option:

- ☐ Modified minimally invasive surgical technique (M-MIST)
- ☐ Minimally invasive surgical technique (MIST)
- ☐ Single flap approach (SFA)
- ☐ Others (Please specify: ____)

b. What biomaterials would you consider using? Please choose only one option:

- ☐ EMD only
- ☐ Bone graft only
- ☐ Resorbable membrane only
- ☐ EMD + bone graft
- ☐ Bone graft + resorbable membrane
- ☐ EMD + bone graft + resorbable membrane
- ☐ No biomaterial
- ☐ Others (Please specify: ____)

End time: ________________

Start time: ________________

44 mesial

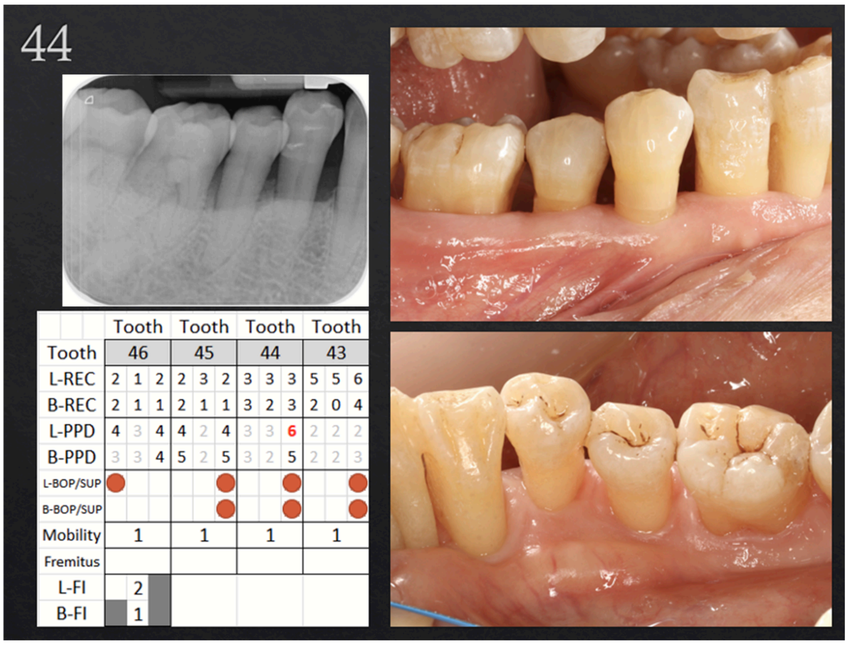

	Tooth			Tooth			Tooth			Tooth		
Tooth	46			45			44			43		
L-REC	2	1	2	2	3	2	3	3	3	5	5	6
B-REC	2	1	1	2	1	1	3	2	3	2	0	4
L-PPD	4	3	4	4	2	4	3	3	6	2	2	2
B-PPD	3	3	4	5	2	5	3	2	5	2	2	3
L-BOP/SUP	●					●			●			●
B-BOP/SUP						●			●			●
Mobility	1			1			1			1		
Fremitus												
L-FI		2										
B-FI		1										

The periapical radiograph above is scaled to actual size. Please perform your depth measurement on this radiograph.

The periapical radiograph below is magnified for better viewing. You may perform your angle measurement on this radiograph.

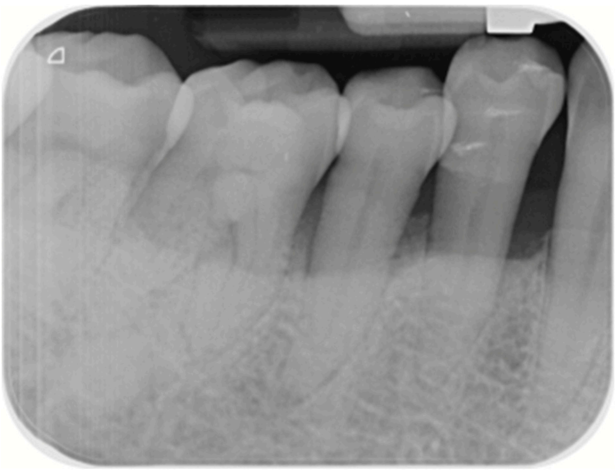

Please enter the start time for this case questionnaire at the top left corner.

1. **Are you currently a:**
   ☐ Second-year resident
   ☐ Third-year resident
   ☐ Resident in higher training
   ☐ Specialist

2. **Does the defect involve:**
   a. More than 1 surface of the root of the same tooth? ☐ Yes ☐ No
   [If you answered "No" for a), please proceed to Q3]
   b. If you answered "Yes" for a), which surfaces of the root does the defect involve? You may choose more than one answer:
   ☐ Mesial and buccal
   ☐ Mesial and lingual
   ☐ Mesial - buccal - distal
   ☐ Mesial - lingual - distal
   ☐ Circumferential

3. **Is there a presence of the following type(s) of infrabony defect? You may choose more than one answer:**
   ☐ 1-walled component
   ☐ 2-walled component
   ☐ 3-walled component
   ☐ Crater
   ☐ Circumferential defect

4. **Is the depth of the defect ≥ 3mm?**
   ☐ Yes ☐ No

5. **Is the angle of the defect ≤ 22 degrees?**
   ☐ Yes ☐ No

6. **Is the angle of the defect ≥ 36 degrees?**
   ☐ Yes ☐ No

7. **Simply considering the defect presentation alone, which procedure do you think is the most appropriate for site management? Please choose only one option:**
   ☐ Non-surgical maintenance
   ☐ Access flap
   ☐ Access flap with osseous resective surgery
   ☐ Periodontal regeneration
   ☐ Tooth extraction
   ☐ Others (Please specify: ____)

8. If periodontal regeneration is selected for Question 7:

a. Which regenerative approach would you select? Please choose only one option:

☐ Modified minimally invasive surgical technique (M-MIST)
☐ Minimally invasive surgical technique (MIST)
☐ Single flap approach (SFA)
☐ Others (Please specify: ____)

b. What biomaterials would you consider using? Please choose only one option:

☐ EMD only
☐ Bone graft only
☐ Resorbable membrane only
☐ EMD + bone graft
☐ Bone graft + resorbable membrane
☐ EMD + bone graft + resorbable membrane
☐ No biomaterial
☐ Others (Please specify: ____)

End time: ________________

Start time: ________________

34 mesial

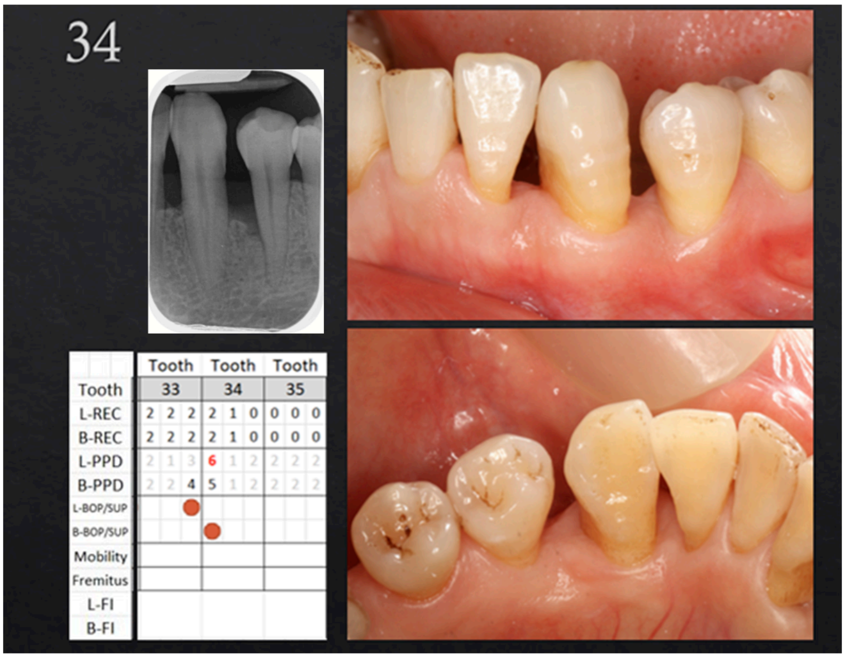

The periapical radiograph above is scaled to actual size. Please perform your depth measurement on this radiograph.

The periapical radiograph below is magnified for better viewing. You may perform your angle measurement on this radiograph.

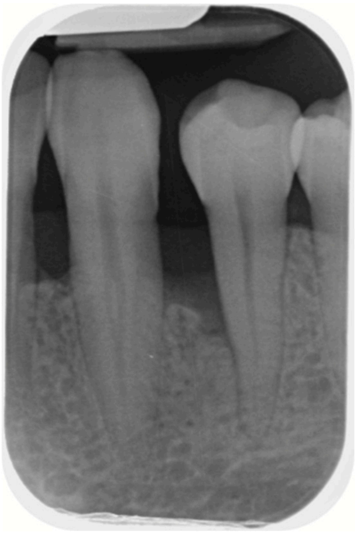

Please enter the start time for this case questionnaire at the top left corner.

1. **Are you currently a:**
   ☐ Second-year resident
   ☐ Third-year resident
   ☐ Resident in higher training
   ☐ Specialist

2. **Does the defect involve:**
   a. More than 1 surface of the root of the same tooth? ☐ Yes ☐ No
   [If you answered "No" for a), please proceed to Q3]
   b. If you answered "Yes" for a), which surfaces of the root does the defect involve? You may choose more than one answer:
   ☐ Mesial and buccal
   ☐ Mesial and lingual
   ☐ Mesial - buccal - distal
   ☐ Mesial - lingual - distal
   ☐ Circumferential

3. **Is there a presence of the following type(s) of infrabony defect? You may choose more than one answer:**
   ☐ 1-walled component
   ☐ 2-walled component
   ☐ 3-walled component
   ☐ Crater
   ☐ Circumferential defect

4. **Is the depth of the defect ≥ 3mm?**
   ☐ Yes ☐ No

5. **Is the angle of the defect ≤ 22 degrees?**
   ☐ Yes ☐ No

6. **Is the angle of the defect ≥ 36 degrees?**
   ☐ Yes ☐ No

7. **Simply considering the defect presentation alone, which procedure do you think is the most appropriate for site management? Please choose only one option:**
   ☐ Non-surgical maintenance
   ☐ Access flap
   ☐ Access flap with osseous resective surgery
   ☐ Periodontal regeneration
   ☐ Tooth extraction
   ☐ Others (Please specify: ____)

8. If periodontal regeneration is selected for Question 7:

a. Which regenerative approach would you select? Please choose only one option:

☐ Modified minimally invasive surgical technique (M-MIST)
☐ Minimally invasive surgical technique (MIST)
☐ Single flap approach (SFA)
☐ Others (Please specify: ____)

b. What biomaterials would you consider using? Please choose only one option:

☐ EMD only
☐ Bone graft only
☐ Resorbable membrane only
☐ EMD + bone graft
☐ Bone graft + resorbable membrane
☐ EMD + bone graft + resorbable membrane
☐ No biomaterial
☐ Others (Please specify: ____)

End time: ________________

[Case 3] Participant Number: 40

Start time: ________________

43 mesial

43

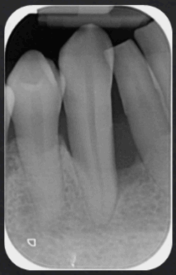

	Tooth			Tooth			Missing			Tooth		
Tooth	44			43			0			41		
L-REC	2	2	1	3	5	3				1	2	2
B-REC	1	2	1	1	3	4				2	3	2
L-PPD	2	2	3	5	4	7				4	2	3
B-PPD	2	2	3	2	2	8				2	1	2
L-BOP/SUP	●			●	●	●						
B-BOP/SUP						●						
Mobility										1		
Fremitus												
L-FI												
B-FI												

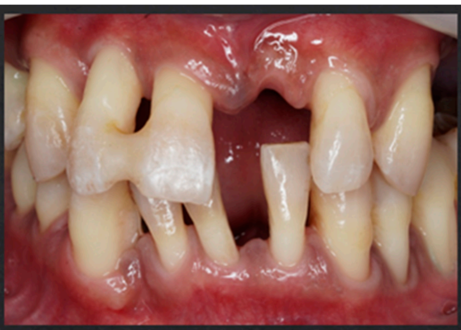

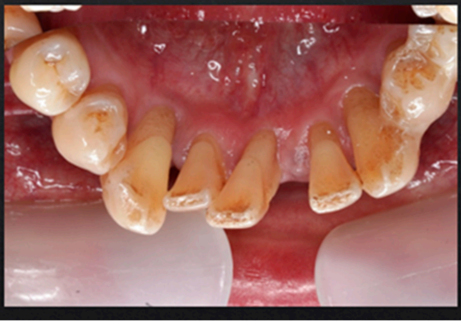

The periapical radiograph above is scaled to actual size. Please perform your depth measurement on this radiograph.

The periapical radiograph below is magnified for better viewing. You may perform your angle measurement on this radiograph.

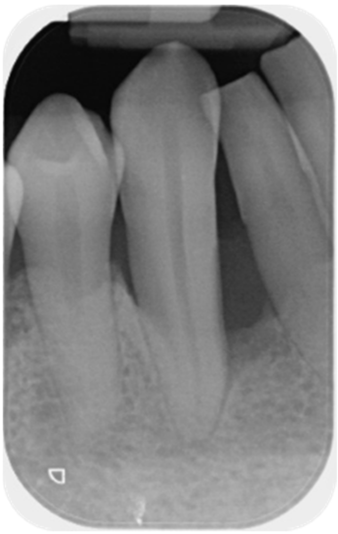

Please enter the start time for this case questionnaire at the top left corner.

1. **Are you currently a:**
   ☐ Second-year resident
   ☐ Third-year resident
   ☐ Resident in higher training
   ☐ Specialist

2. **Does the defect involve:**
   a. More than 1 surface of the root of the same tooth? ☐ Yes ☐ No
   [If you answered "No" for a), please proceed to Q3]
   b. If you answered "Yes" for a), which surfaces of the root does the defect involve? You may choose more than one answer:
   ☐ Mesial and buccal
   ☐ Mesial and lingual
   ☐ Mesial - buccal - distal
   ☐ Mesial - lingual - distal
   ☐ Circumferential

3. **Is there a presence of the following type(s) of infrabony defect? You may choose more than one answer:**
   ☐ 1-walled component
   ☐ 2-walled component
   ☐ 3-walled component
   ☐ Crater
   ☐ Circumferential defect

4. **Is the depth of the defect ≥ 3mm?**
   ☐ Yes ☐ No

5. **Is the angle of the defect ≤ 22 degrees?**
   ☐ Yes ☐ No

6. **Is the angle of the defect ≥ 36 degrees?**
   ☐ Yes ☐ No

7. **Simply considering the defect presentation alone, which procedure do you think is the most appropriate for site management? Please choose only one option:**
   ☐ Non-surgical maintenance
   ☐ Access flap
   ☐ Access flap with osseous resective surgery
   ☐ Periodontal regeneration
   ☐ Tooth extraction
   ☐ Others (Please specify: ____)

8. If periodontal regeneration is selected for Question 7:

a. Which regenerative approach would you select? Please choose only one option:

☐ Modified minimally invasive surgical technique (M-MIST)
☐ Minimally invasive surgical technique (MIST)
☐ Single flap approach (SFA)
☐ Others (Please specify: ____)

b. What biomaterials would you consider using? Please choose only one option:

☐ EMD only
☐ Bone graft only
☐ Resorbable membrane only
☐ EMD + bone graft
☐ Bone graft + resorbable membrane
☐ EMD + bone graft + resorbable membrane
☐ No biomaterial
☐ Others (Please specify: ____)

End time: ________________

[Case 6] Participant Number: 40

Start time: ________________

43 distal

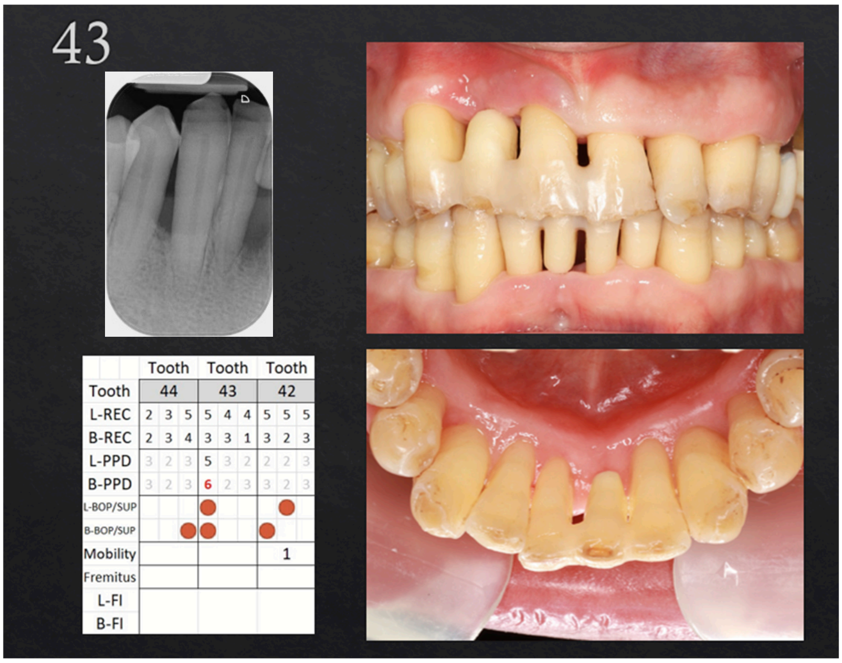

	Tooth			Tooth			Tooth		
Tooth	44			43			42		
L-REC	2	3	5	5	4	4	5	5	5
B-REC	2	3	4	3	3	1	3	2	3
L-PPD	3	2	3	5	3	2	2	2	3
B-PPD	3	2	3	6	2	3	3	2	3
L-BOP/SUP				●				●	
B-BOP/SUP			●	●			●		
Mobility								1	
Fremitus									
L-FI									
B-FI									

The periapical radiograph above is scaled to actual size. Please perform your depth measurement on this radiograph.

The periapical radiograph below is magnified for better viewing. You may perform your angle measurement on this radiograph.

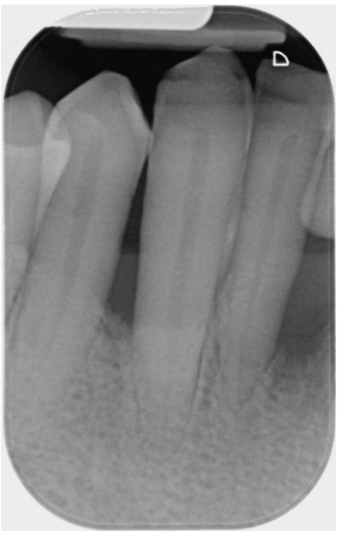

Please enter the start time for this case questionnaire at the top left corner.

1. **Are you currently a:**
   ☐ Second-year resident
   ☐ Third-year resident
   ☐ Resident in higher training
   ☐ Specialist

2. **Does the defect involve:**
   a. More than 1 surface of the root of the same tooth? ☐ Yes ☐ No
   [If you answered "No" for a), please proceed to Q3]
   b. If you answered "Yes" for a), which surfaces of the root does the defect involve? You may choose more than one answer:
   ☐ Distal and buccal
   ☐ Distal and lingual
   ☐ Distal - buccal - mesial
   ☐ Distal - lingual - mesial
   ☐ Circumferential

3. **Is there a presence of the following type(s) of infrabony defect? You may choose more than one answer:**
   ☐ 1-walled component
   ☐ 2-walled component
   ☐ 3-walled component
   ☐ Crater
   ☐ Circumferential defect

4. **Is the depth of the defect ≥ 3mm?**
   ☐ Yes ☐ No

5. **Is the angle of the defect ≤ 22 degrees?**
   ☐ Yes ☐ No

6. **Is the angle of the defect ≥ 36 degrees?**
   ☐ Yes ☐ No

7. **Simply considering the defect presentation alone, which procedure do you think is the most appropriate for site management? Please choose only one option:**
   ☐ Non-surgical maintenance
   ☐ Access flap
   ☐ Access flap with osseous resective surgery
   ☐ Periodontal regeneration
   ☐ Tooth extraction
   ☐ Others (Please specify: ____)

8. If periodontal regeneration is selected for Question 7:

a. Which regenerative approach would you select? Please choose only one option:

☐ Modified minimally invasive surgical technique (M-MIST)
☐ Minimally invasive surgical technique (MIST)
☐ Single flap approach (SFA)
☐ Others (Please specify: ____)

b. What biomaterials would you consider using? Please choose only one option:

☐ EMD only
☐ Bone graft only
☐ Resorbable membrane only
☐ EMD + bone graft
☐ Bone graft + resorbable membrane
☐ EMD + bone graft + resorbable membrane
☐ No biomaterial
☐ Others (Please specify: ____)

End time: ________________

Start time: ________________

14 mesial

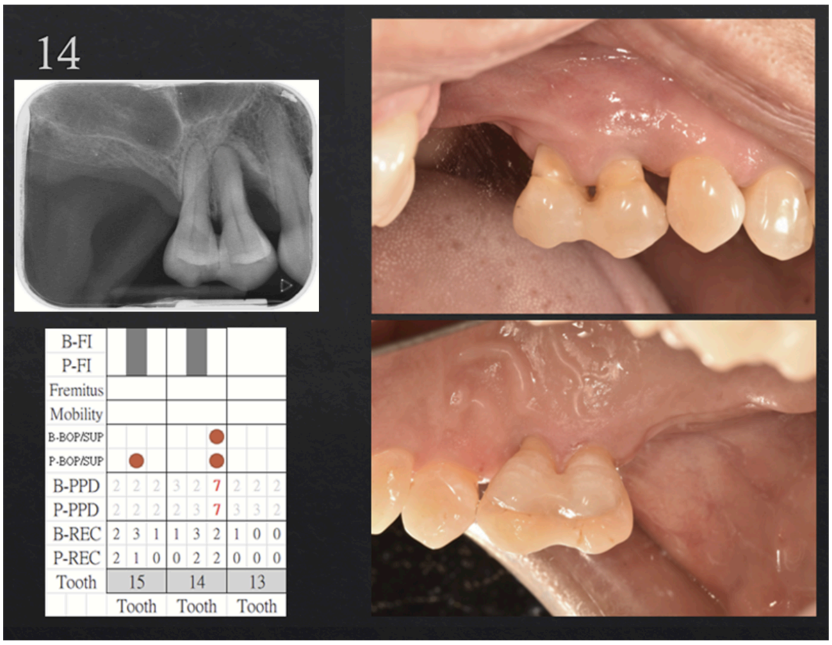

	15			14			13		
B-FI									
P-FI									
Fremitus									
Mobility									
B-BOP/SUP						●			
P-BOP/SUP		●				●			
B-PPD	2	2	2	3	2	7	2	2	2
P-PPD	2	2	2	2	3	7	3	3	2
B-REC	2	3	1	1	3	2	1	0	0
P-REC	2	1	0	0	2	2	0	0	0
Tooth	15			14			13		
	Tooth			Tooth			Tooth		

The periapical radiograph above is scaled to actual size. Please perform your depth measurement on this radiograph.

The periapical radiograph below is magnified for better viewing. You may perform your angle measurement on this radiograph.

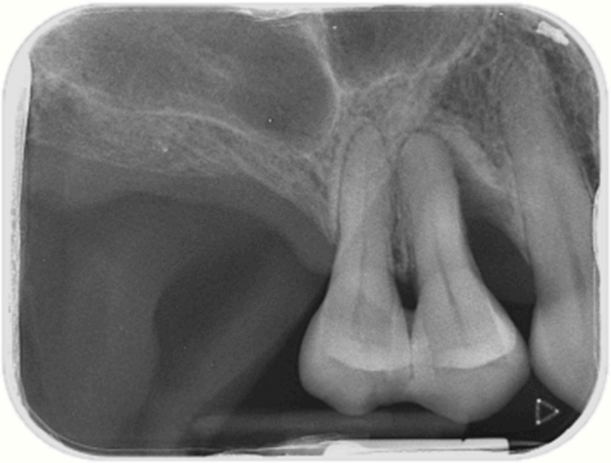

Please enter the start time for this case questionnaire at the top left corner.

1. **Are you currently a:**
   ☐ Second-year resident
   ☐ Third-year resident
   ☐ Resident in higher training
   ☐ Specialist

2. **Does the defect involve:**
   a. More than 1 surface of the root of the same tooth? ☐ Yes ☐ No
   [If you answered "No" for a), please proceed to Q3]
   b. If you answered "Yes" for a), which surfaces of the root does the defect involve? You may choose more than one answer:
   ☐ Mesial and buccal
   ☐ Mesial and palatal
   ☐ Mesial - buccal - distal
   ☐ Mesial - palatal - distal
   ☐ Circumferential

3. **Is there a presence of the following type(s) of infrabony defect? You may choose more than one answer:**
   ☐ 1-walled component
   ☐ 2-walled component
   ☐ 3-walled component
   ☐ Crater
   ☐ Circumferential defect

4. **Is the depth of the defect ≥ 3mm?**
   ☐ Yes ☐ No

5. **Is the angle of the defect ≤ 22 degrees?**
   ☐ Yes ☐ No

6. **Is the angle of the defect ≥ 36 degrees?**
   ☐ Yes ☐ No

7. **Simply considering the defect presentation alone, which procedure do you think is the most appropriate for site management? Please choose only one option:**
   ☐ Non-surgical maintenance
   ☐ Access flap
   ☐ Access flap with osseous resective surgery
   ☐ Periodontal regeneration
   ☐ Tooth extraction
   ☐ Others (Please specify: ____)

8. If periodontal regeneration is selected for Question 7:

a. Which regenerative approach would you select? Please choose only one option:

☐ Modified minimally invasive surgical technique (M-MIST)
☐ Minimally invasive surgical technique (MIST)
☐ Single flap approach (SFA)
☐ Others (Please specify: ____)

b. What biomaterials would you consider using? Please choose only one option:

☐ EMD only
☐ Bone graft only
☐ Resorbable membrane only
☐ EMD + bone graft
☐ Bone graft + resorbable membrane
☐ EMD + bone graft + resorbable membrane
☐ No biomaterial
☐ Others (Please specify: ____)

End time: ________________

Start time: ________________

12 distal

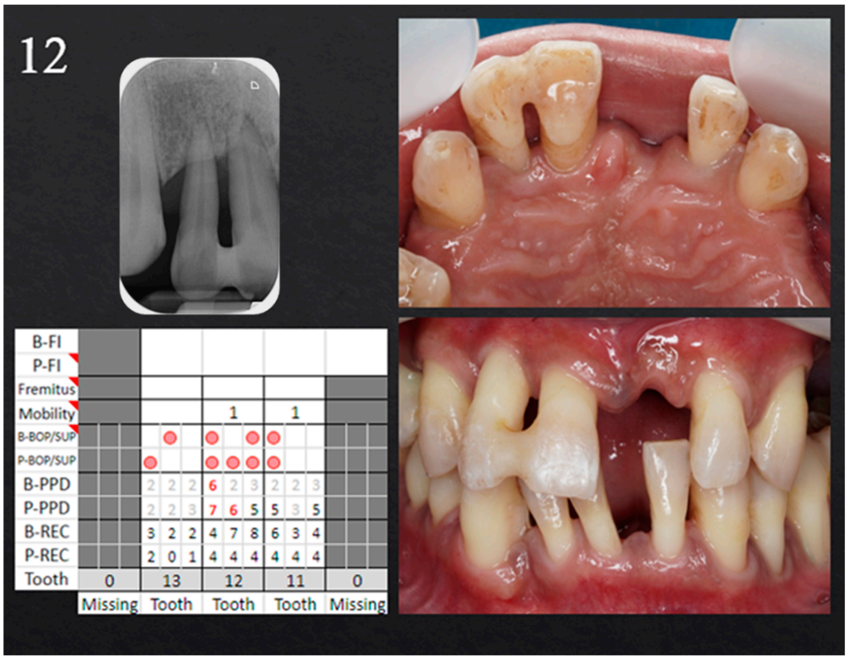

The periapical above radiograph is scaled to actual size. Please perform your depth measurement on this radiograph.

The periapical radiograph below is magnified for better viewing. You may perform your angle measurement on this radiograph.

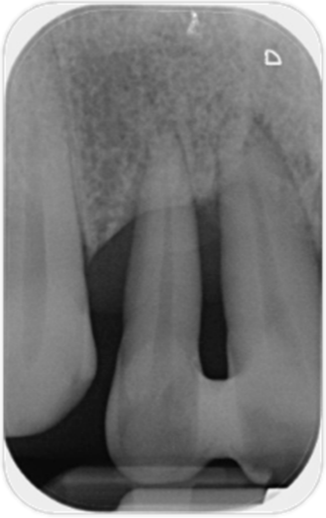

Please enter the start time for this case questionnaire at the top left corner.

1. **Are you currently a:**
   ☐ Second-year resident
   ☐ Third-year resident
   ☐ Resident in higher training
   ☐ Specialist

2. **Does the defect involve:**
   a. More than 1 surface of the root of the same tooth? ☐ Yes ☐ No
   [If you answered "No" for a), please proceed to Q3]
   b. If you answered "Yes" for a), which surfaces of the root does the defect involve? You may choose more than one answer:
   ☐ Distal and buccal
   ☐ Distal and palatal
   ☐ Distal - buccal - mesial
   ☐ Distal - palatal - mesial
   ☐ Circumferential

3. **Is there a presence of the following type(s) of infrabony defect? You may choose more than one answer:**
   ☐ 1-walled component
   ☐ 2-walled component
   ☐ 3-walled component
   ☐ Crater
   ☐ Circumferential defect

4. **Is the depth of the defect ≥ 3mm?**
   ☐ Yes ☐ No

5. **Is the angle of the defect ≤ 22 degrees?**
   ☐ Yes ☐ No

6. **Is the angle of the defect ≥ 36 degrees?**
   ☐ Yes ☐ No

7. **Simply considering the defect presentation alone, which procedure do you think is the most appropriate for site management? Please choose only one option:**
   ☐ Non-surgical maintenance
   ☐ Access flap
   ☐ Access flap with osseous resective surgery
   ☐ Periodontal regeneration
   ☐ Tooth extraction
   ☐ Others (Please specify: ____)

8. If periodontal regeneration is selected for Question 7:

a. Which regenerative approach would you select? Please choose only one option:

☐ Modified minimally invasive surgical technique (M-MIST)
☐ Minimally invasive surgical technique (MIST)
☐ Single flap approach (SFA)
☐ Others (Please specify: ____)

b. What biomaterials would you consider using? Please choose only one option:

☐ EMD only
☐ Bone graft only
☐ Resorbable membrane only
☐ EMD + bone graft
☐ Bone graft + resorbable membrane
☐ EMD + bone graft + resorbable membrane
☐ No biomaterial
☐ Others (Please specify: ____)

End time: ________________

Start time: ________________

41 distal

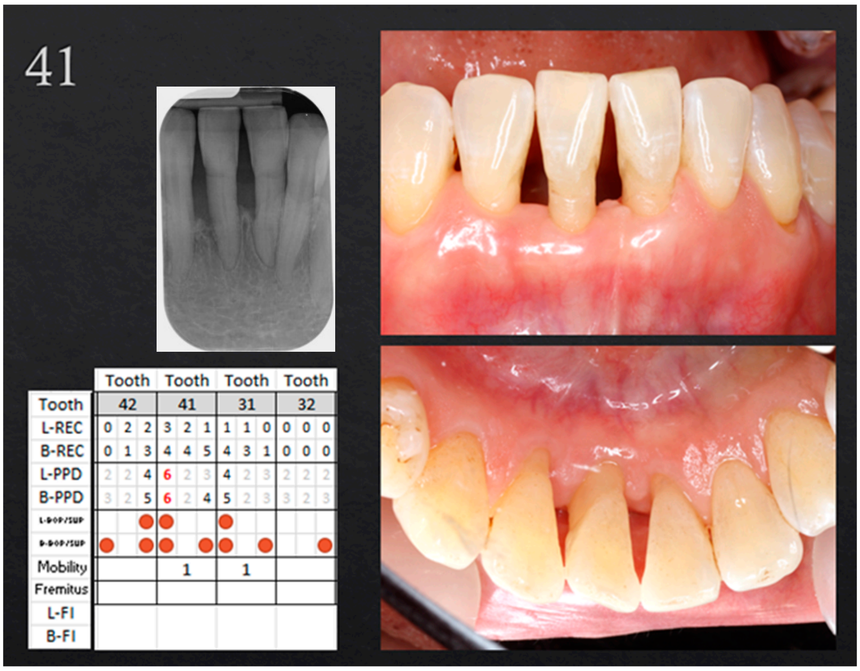

	Tooth			Tooth			Tooth			Tooth		
Tooth	42			41			31			32		
L-REC	0	2	2	3	2	1	1	1	0	0	0	0
B-REC	0	1	3	4	4	5	4	3	1	0	0	0
L-PPD	2	2	4	6	2	3	4	2	3	2	2	2
B-PPD	3	2	5	6	2	4	5	2	3	3	2	3
L-BOP/SUP			●	●			●					
B-BOP/SUP	●		●	●		●	●		●			●
Mobility		1					1					
Fremitus												
L-FI												
B-FI												

The periapical radiograph above is scaled to actual size. Please perform your depth measurement on this radiograph.

The periapical radiograph below is magnified for better viewing. You may perform your angle measurement on this radiograph.

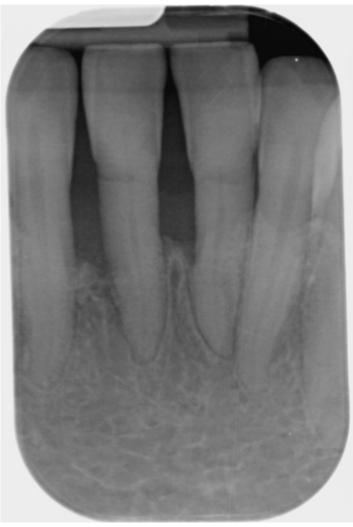

Please enter the start time for this case questionnaire at the top left corner.

1. **Are you currently a:**
   ☐ Second-year resident
   ☐ Third-year resident
   ☐ Resident in higher training
   ☐ Specialist

2. **Does the defect involve:**
   a. More than 1 surface of the root of the same tooth? ☐ Yes ☐ No
   [If you answered "No" for a), please proceed to Q3]
   b. If you answered "Yes" for a), which surfaces of the root does the defect involve? You may choose more than one answer:
   ☐ Distal and buccal
   ☐ Distal and lingual
   ☐ Distal - buccal - mesial
   ☐ Distal - lingual - mesial
   ☐ Circumferential

3. **Is there a presence of the following type(s) of infrabony defect? You may choose more than one answer:**
   ☐ 1-walled component
   ☐ 2-walled component
   ☐ 3-walled component
   ☐ Crater
   ☐ Circumferential defect

4. **Is the depth of the defect ≥ 3mm?**
   ☐ Yes ☐ No

5. **Is the angle of the defect ≤ 22 degrees?**
   ☐ Yes ☐ No

6. **Is the angle of the defect ≥ 36 degrees?**
   ☐ Yes ☐ No

7. **Simply considering the defect presentation alone, which procedure do you think is the most appropriate for site management? Please choose only one option:**
   ☐ Non-surgical maintenance
   ☐ Access flap
   ☐ Access flap with osseous resective surgery
   ☐ Periodontal regeneration
   ☐ Tooth extraction
   ☐ Others (Please specify: ____)

8. If periodontal regeneration is selected for Question 7:

a. Which regenerative approach would you select? Please choose only one option:

☐ Modified minimally invasive surgical technique (M-MIST)
☐ Minimally invasive surgical technique (MIST)
☐ Single flap approach (SFA)
☐ Others (Please specify: ____)

b. What biomaterials would you consider using? Please choose only one option:

☐ EMD only
☐ Bone graft only
☐ Resorbable membrane only
☐ EMD + bone graft
☐ Bone graft + resorbable membrane
☐ EMD + bone graft + resorbable membrane
☐ No biomaterial
☐ Others (Please specify: ____)

End time: ________________

Questionnaire S2

Questionnaire S2 contains 8 cases with completion of Step 1 & 2 therapy. In each case, the location of the periodontal defect is specified.

The following information is given for each case:

- *Periodontal Re-evaluation clinical and radiographic data:*
    - *Periodontal charting*
        - *Parameters:*
            - *6-point PPD, recession, BOP*
            - *Furcation involvement (Hamp et al.)*
            - *Tooth hypermobility (Miller)*
            - *Presence of fremitus*
    - *Periapical radiograph(s) (scaled to actual size)*
    - *Intra-oral photographs*

- *CBCT data presented in images (magnified for ease of viewing):*
    - *A fixed axial view (containing multiple axial slices of thickness 0.2mm, separated at 0.5mm intervals)*
    - *A fixed coronal view*
    - *A fixed sagittal view*
    - *A fixed 3D rendering image*

Participant Number: 40

Please calculate the depth of the infrabony defect from the CBCT axial slices provided. Please use the following formula:

Defect depth = (number of axial slices involving the defect -1) x 0.5mm + (0.2mm / 2) x 2

- *Number of axial slices involving the defect -1 = number of slice intervals involved*
- *0.5mm is the slice interval*
- *0.2mm is the slice thickness*
    - *0.2mm is divided by 2, as the slice interval bisects the thickness of the most coronal and apical axial slice of the defect*
    - *The bisected thickness (0.1mm) is then multiplied by 2 to include both the most coronal and apical axial slice of the defect in the defect depth*

Here is a sample calculation for the infrabony defect at 16 mesial:

Defect depth = (slices #4 to #11 -1) x 0.5mm + (0.2mm / 2) x 2
= (8-1) slices x 0.5mm + (0.2mm / 2) x 2
= 3.5mm + (0.2mm / 2) x 2
= 3.5mm + 0.1mm x 2
= 3.7mm

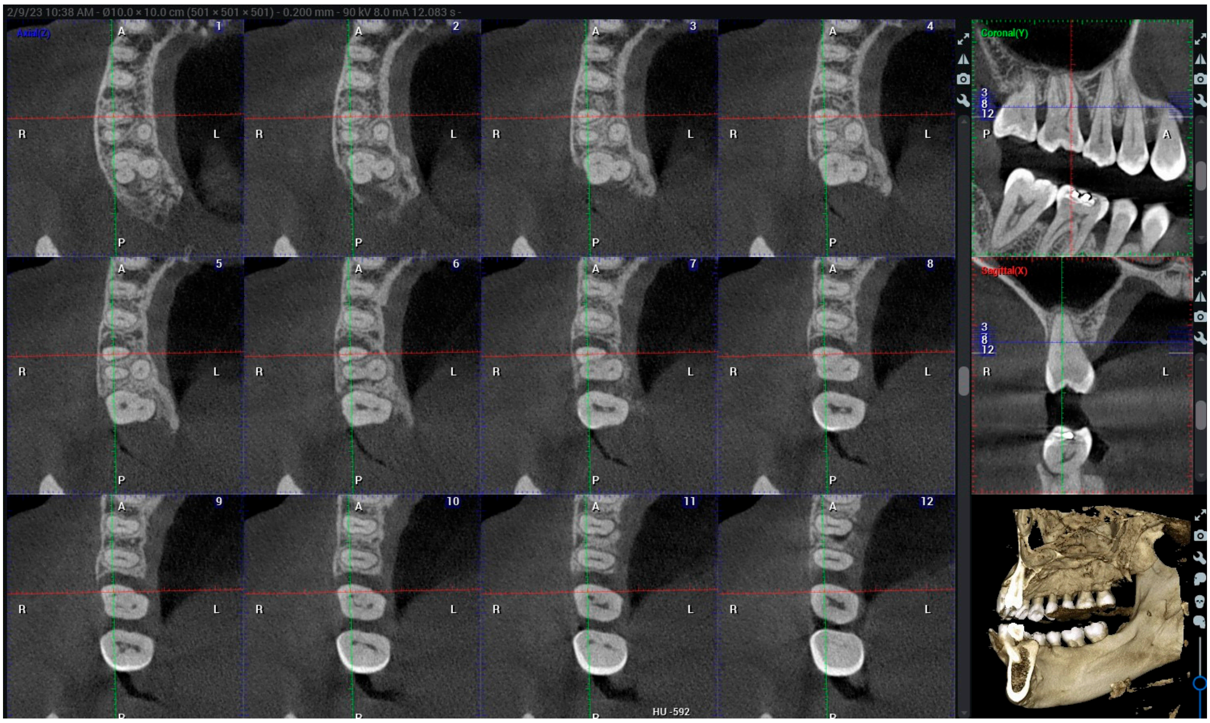

Please measure the angle of the infrabony defect on the CBCT sagittal slice provided. Please use the following method:

*Angle of defect = the angle formed by the two lines **AC**-**BD** and **BD**-**CEJ**, where:*

- ***BD**-**CEJ** = the root surface of the involved tooth*
- ***AC**-**BD** = the bone defect surface*

Here is a measurement demonstration for the infrabony defect at 16 mesial:

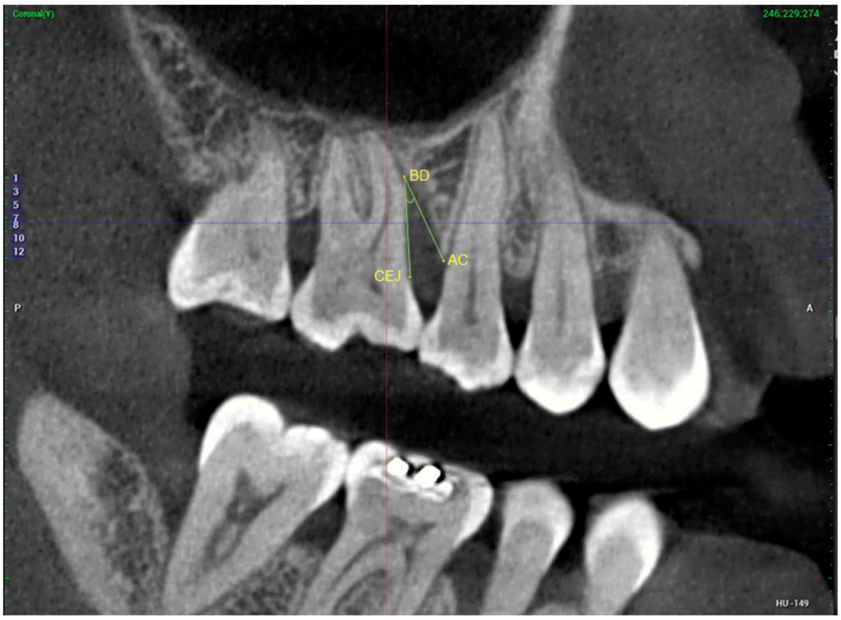

*Finally, please kindly complete all the cases and **note down the start and end time** for each case.*

Thank you for your participation.

Start time: ________________

44 mesial

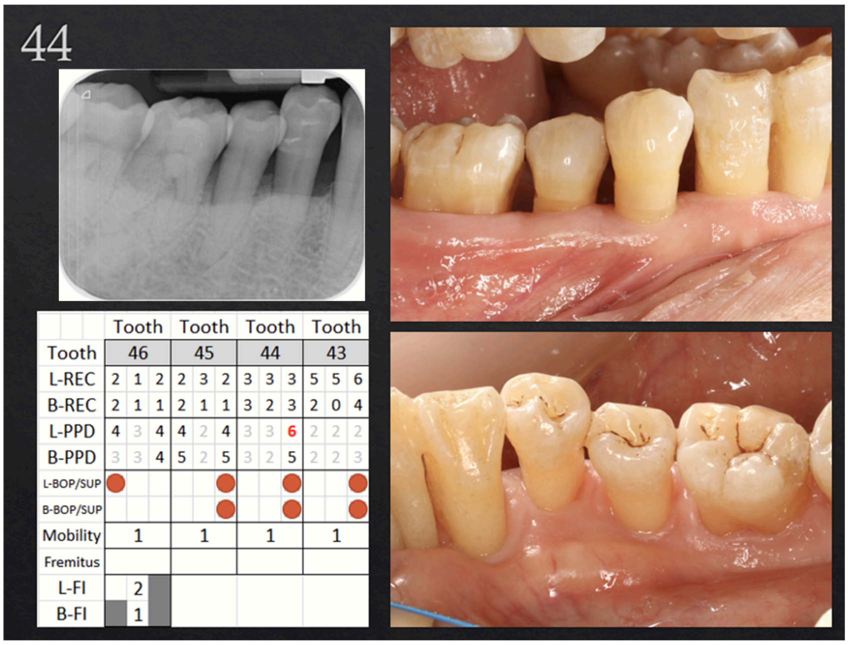

	Tooth			Tooth			Tooth			Tooth		
Tooth	46			45			44			43		
L-REC	2	1	2	2	3	2	3	3	3	5	5	6
B-REC	2	1	1	2	1	1	3	2	3	2	0	4
L-PPD	4	3	4	4	2	4	3	3	6	2	2	2
B-PPD	3	3	4	5	2	5	3	2	5	2	2	3
L-BOP/SUP	●					●			●			●
B-BOP/SUP						●			●			●
Mobility	1			1			1			1		
Fremitus												
L-FI		2										
B-FI		1										

The periapical radiograph is scaled to actual size.

Please enter the start time for this case questionnaire at the top left corner.

1. **Are you currently a:**
   - ☐ Second-year resident
   - ☐ Third-year resident
   - ☐ Resident in higher training
   - ☐ Specialist

Please find a fixed CBCT axial view of the defect below. Each slice is of 0.2mm thickness. The interval between each slice is 0.5mm. Please note that the slices are magnified for ease of viewing.

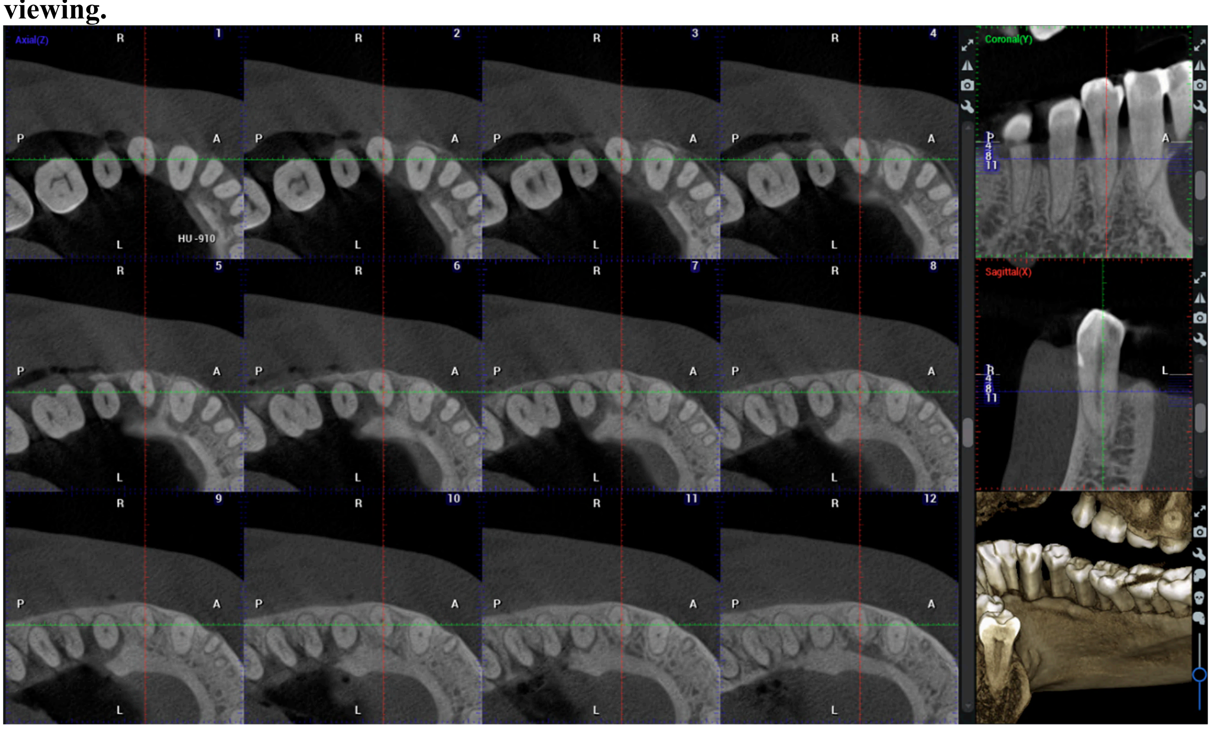

2. **Does the defect involve:**
   a. More than 1 surface of the root of the same tooth? ☐ Yes ☐ No
      [If you answered "No" for a), please proceed to Q3]
   b. If you answered "Yes" for a), which surfaces of the root does the defect involve? You may choose more than one answer:
      ☐ Mesial and buccal
      ☐ Mesial and lingual
      ☐ Mesial - buccal - distal
      ☐ Mesial - lingual - distal
      ☐ Circumferential

3. **Is there a presence of the following type(s) of infrabony defect? You may choose more than one answer:**
   ☐ 1-walled component
   ☐ 2-walled component
   ☐ 3-walled component
   ☐ Crater
   ☐ Circumferential defect

4. **Is the depth of the defect ≥ 3mm?**
   [Defect depth = (number of axial slices involving the defect -1) x 0.5mm + (0.2mm / 2) x 2]
   ☐ Yes ☐ No

Please find a fixed CBCT sagittal view of the defect below. The slice is magnified for ease of viewing.

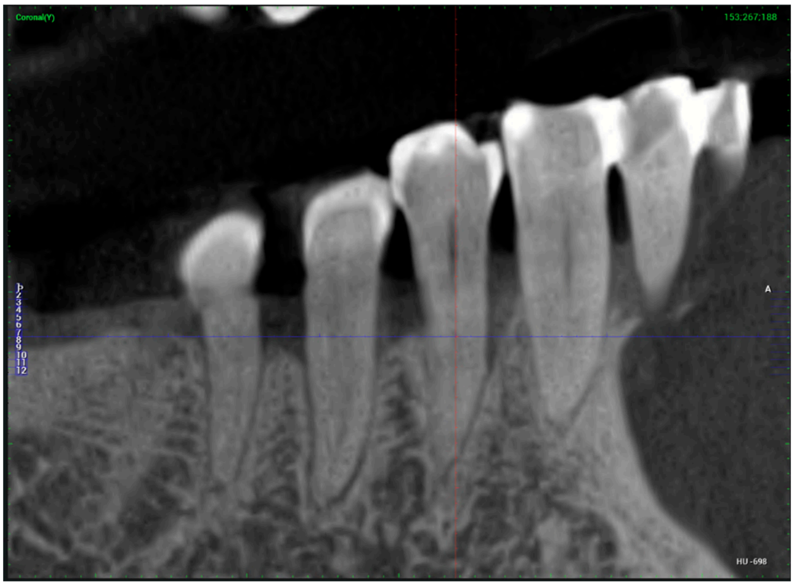

5. **Is the angle of the defect ≤ 22 degrees?**
   ☐ Yes ☐ No

6. **Is the angle of the defect ≥ 36 degrees?**
   ☐ Yes ☐ No

7. **Simply considering the defect presentation alone, which procedure do you think is the most appropriate for site management? Please choose only one option:**
   - ☐ Non-surgical maintenance
   - ☐ Access flap
   - ☐ Access flap with osseous resective surgery
   - ☐ Periodontal regeneration
   - ☐ Tooth extraction
   - ☐ Others (Please specify: ______________________________)

8. **If periodontal regeneration is selected for Question 7:**
   a. **Which regenerative approach would you select? Please choose only one option:**
   - ☐ Modified minimally invasive surgical technique (M-MIST)
   - ☐ Minimally invasive surgical technique (MIST)
   - ☐ Single flap approach (SFA)
   - ☐ Others (Please specify: ______________________________)

   b. **What biomaterials would you consider using? Please choose only one option:**
   - ☐ EMD only
   - ☐ Bone graft only
   - ☐ Resorbable membrane only
   - ☐ EMD + bone graft
   - ☐ Bone graft + resorbable membrane
   - ☐ EMD + bone graft + resorbable membrane
   - ☐ No biomaterial
   - ☐ Others (Please specify: ______________________________)

End time: ______________

Start time: ________________

12 distal

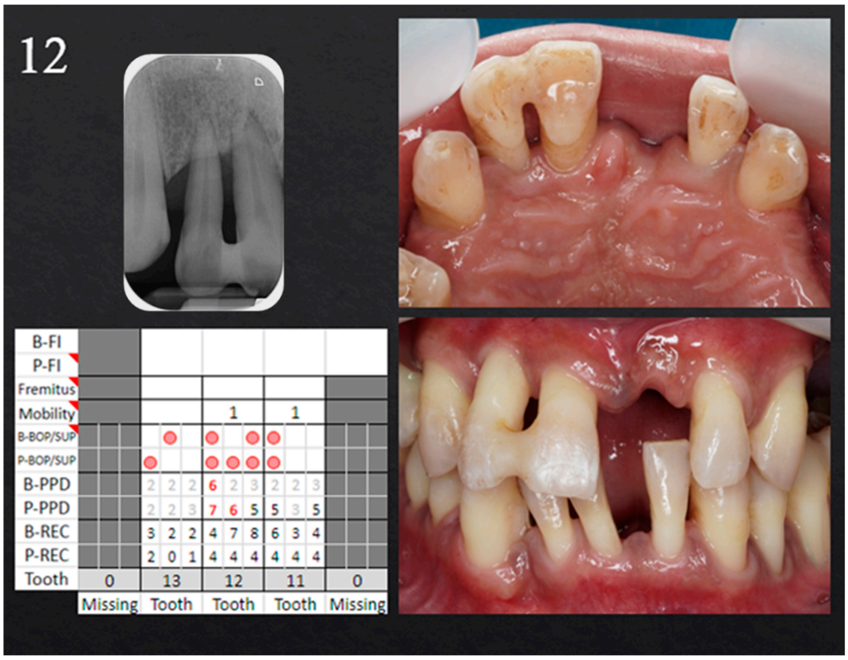

The periapical radiograph is scaled to actual size.

Please enter the start time for this case questionnaire at the top left corner.

1. **Are you currently a:**
   ☐ Second-year resident
   ☐ Third-year resident
   ☐ Resident in higher training
   ☐ Specialist

Please find a fixed CBCT axial view of the defect below. Each slice is of 0.2mm thickness. The interval between each slice is 0.5mm. Please note that the slices are magnified for ease of viewing.

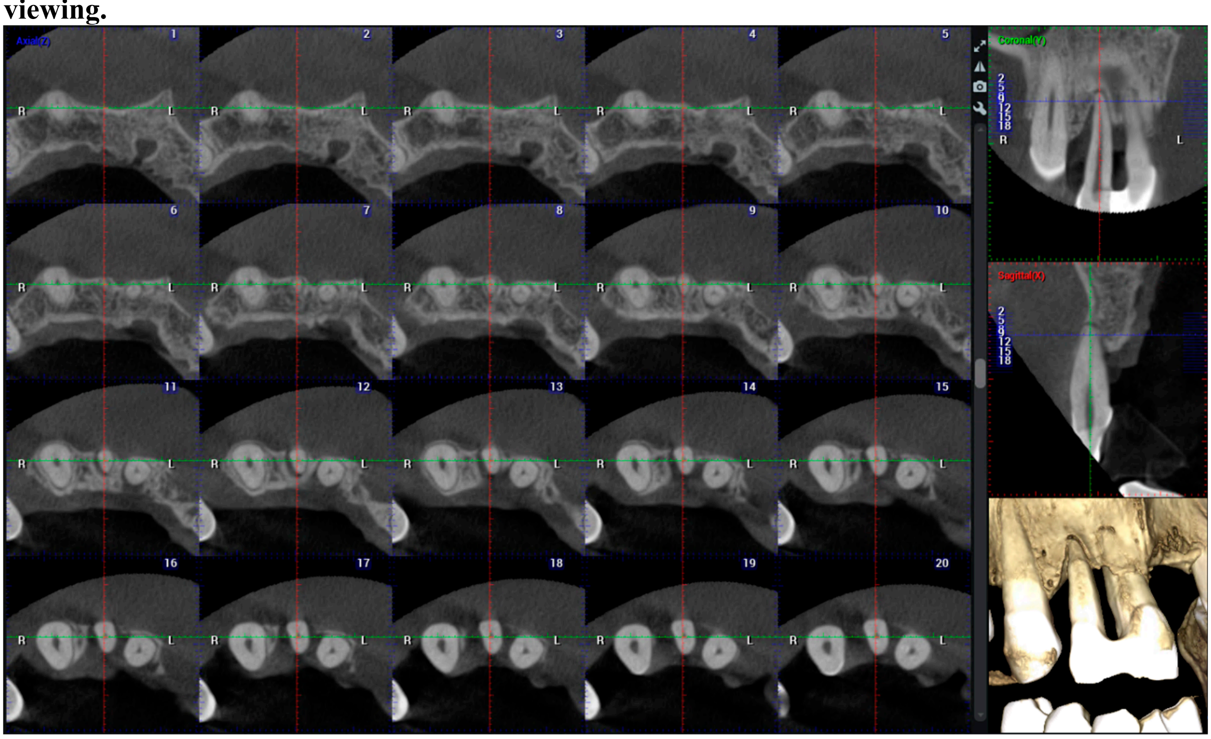

2. **Does the defect involve:**
   a. More than 1 surface of the root of the same tooth? ☐ Yes ☐ No
   [If you answered "No" for a), please proceed to Q3]
   b. If you answered "Yes" for a), which surfaces of the root does the defect involve? You may choose more than one answer:
   ☐ Distal and buccal
   ☐ Distal and palatal
   ☐ Distal - buccal - mesial
   ☐ Distal - palatal - mesial
   ☐ Circumferential

3. **Is there a presence of the following type(s) of infrabony defect? You may choose more than one answer:**
   ☐ 1-walled component
   ☐ 2-walled component
   ☐ 3-walled component
   ☐ Crater
   ☐ Circumferential defect

4. **Is the depth of the defect ≥ 3mm?**
   [Defect depth = (number of axial slices involving the defect -1) x 0.5mm + (0.2mm / 2) x 2]
   ☐ Yes ☐ No

Please find a fixed CBCT sagittal view of the defect below. The slice is magnified for ease of viewing.

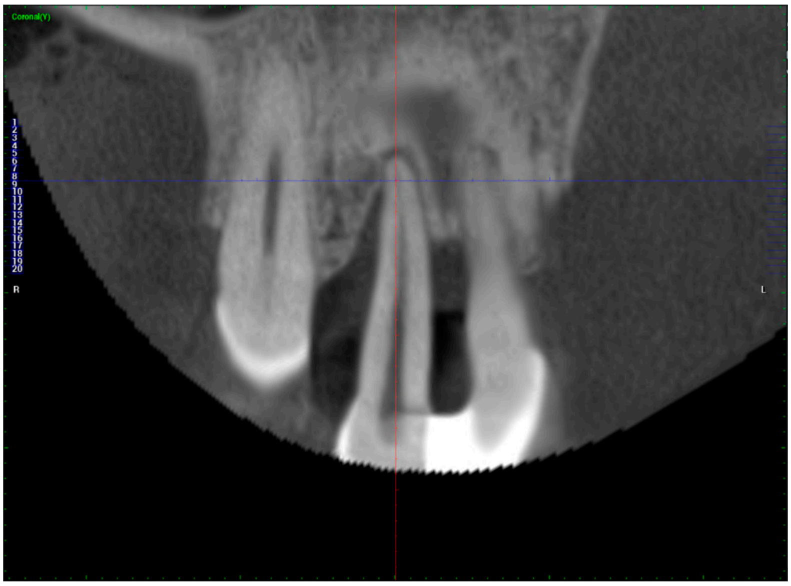

5. **Is the angle of the defect ≤ 22 degrees?**
   ☐ Yes ☐ No

6. **Is the angle of the defect ≥ 36 degrees?**
   ☐ Yes ☐ No

7. **Simply considering the defect presentation alone, which procedure do you think is the most appropriate for site management? Please choose only one option:**
   ☐ Non-surgical maintenance
   ☐ Access flap
   ☐ Access flap with osseous resective surgery
   ☐ Periodontal regeneration
   ☐ Tooth extraction
   ☐ Others (Please specify: ______________________________)

8. **If periodontal regeneration is selected for Question 7:**
   a. **Which regenerative approach would you select? Please choose only one option:**
   ☐ Modified minimally invasive surgical technique (M-MIST)
   ☐ Minimally invasive surgical technique (MIST)
   ☐ Single flap approach (SFA)
   ☐ Others (Please specify: ______________________________)
   b. **What biomaterials would you consider using? Please choose only one option:**
   ☐ EMD only
   ☐ Bone graft only
   ☐ Resorbable membrane only
   ☐ EMD + bone graft
   ☐ Bone graft + resorbable membrane
   ☐ EMD + bone graft + resorbable membrane
   ☐ No biomaterial
   ☐ Others (Please specify: ______________________________)

End time: ________________

Start time: ________________

34 mesial

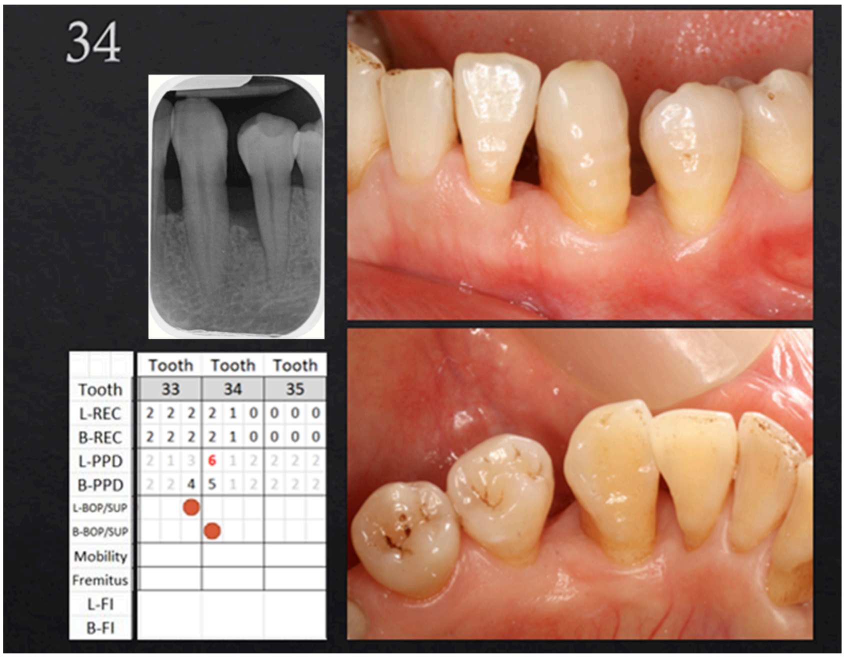

Tooth	Tooth 33			Tooth 34			Tooth 35		
L-REC	2	2	2	2	1	0	0	0	0
B-REC	2	2	2	2	1	0	0	0	0
L-PPD	2	1	3	6	1	2	2	2	2
B-PPD	2	2	4	5	1	2	2	2	2
L-BOP/SUP			●						
B-BOP/SUP				●					
Mobility									
Fremitus									
L-FI									
B-FI									

The periapical radiograph is scaled to actual size.

Please enter the start time for this case questionnaire at the top left corner.

1. **Are you currently a:**
   ☐ Second-year resident
   ☐ Third-year resident
   ☐ Resident in higher training
   ☐ Specialist

Please find a fixed CBCT axial view of the defect below. Each slice is of 0.2mm thickness. The interval between each slice is 0.5mm. Please note that the slices are magnified for ease of viewing.

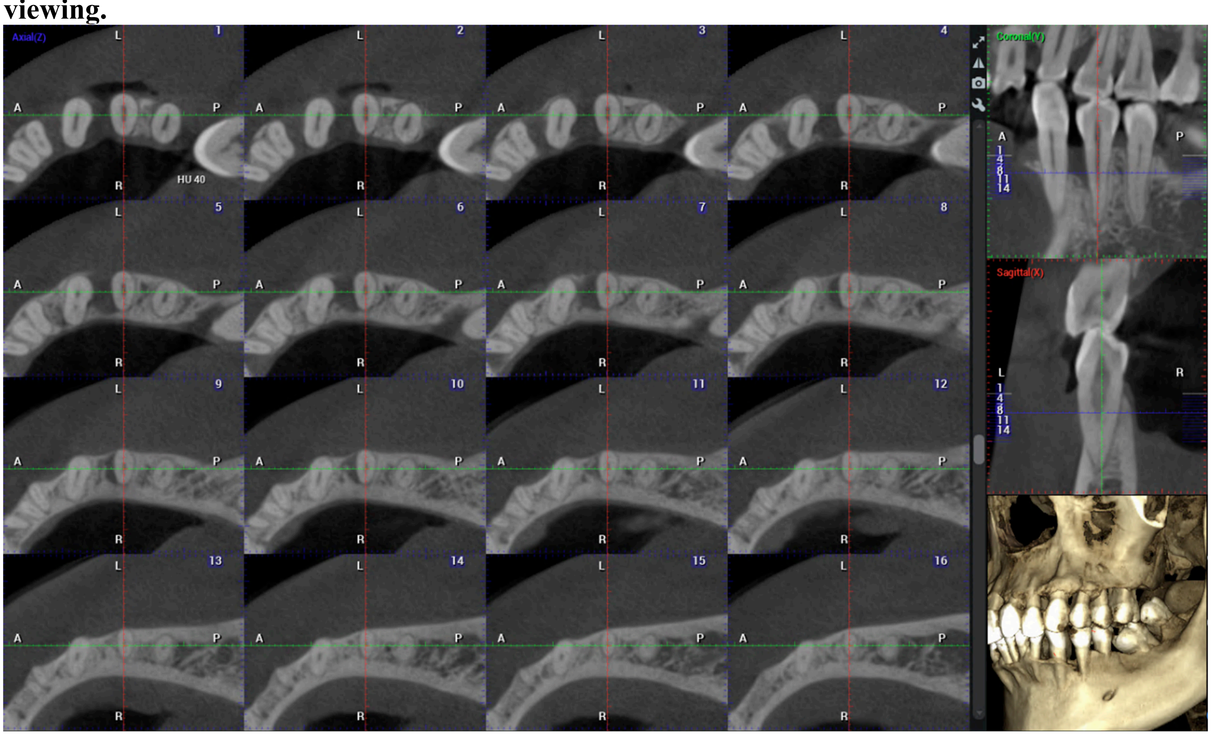

2. **Does the defect involve:**
   a. More than 1 surface of the root of the same tooth? ☐ Yes ☐ No
   [If you answered "No" for a), please proceed to Q3]
   b. If you answered "Yes" for a), which surfaces of the root does the defect involve? You may choose more than one answer:
      ☐ Mesial and buccal
      ☐ Mesial and lingual
      ☐ Mesial - buccal - distal
      ☐ Mesial - lingual - distal
      ☐ Circumferential

3. **Is there a presence of the following type(s) of infrabony defect? You may choose more than one answer:**
   ☐ 1-walled component
   ☐ 2-walled component
   ☐ 3-walled component
   ☐ Crater
   ☐ Circumferential defect

4. **Is the depth of the defect ≥ 3mm?**
   [Defect depth = (number of axial slices involving the defect -1) x 0.5mm + (0.2mm / 2) x 2]
   ☐ Yes ☐ No

Please find a fixed CBCT sagittal view of the defect below. The slice is magnified for ease of viewing.

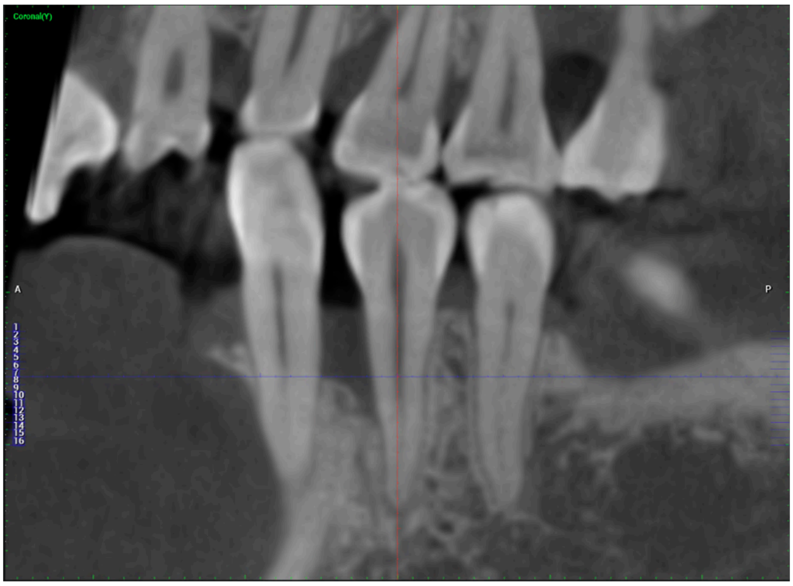

5. **Is the angle of the defect ≤ 22 degrees?**
   ☐ Yes ☐ No

6. **Is the angle of the defect ≥ 36 degrees?**
   ☐ Yes ☐ No

7. **Simply considering the defect presentation alone, which procedure do you think is the most appropriate for site management? Please choose only one option:**
   ☐ Non-surgical maintenance
   ☐ Access flap
   ☐ Access flap with osseous resective surgery
   ☐ Periodontal regeneration
   ☐ Tooth extraction
   ☐ Others (Please specify: ____)

8. **If periodontal regeneration is selected for Question 7:**
   a. **Which regenerative approach would you select? Please choose only one option:**
      ☐ Modified minimally invasive surgical technique (M-MIST)
      ☐ Minimally invasive surgical technique (MIST)
      ☐ Single flap approach (SFA)
      ☐ Others (Please specify: ____)
   b. **What biomaterials would you consider using? Please choose only one option:**
      ☐ EMD only
      ☐ Bone graft only
      ☐ Resorbable membrane only
      ☐ EMD + bone graft
      ☐ Bone graft + resorbable membrane
      ☐ EMD + bone graft + resorbable membrane
      ☐ No biomaterial
      ☐ Others (Please specify: ____)

End time: _______________

Start time: ________________

43 mesial

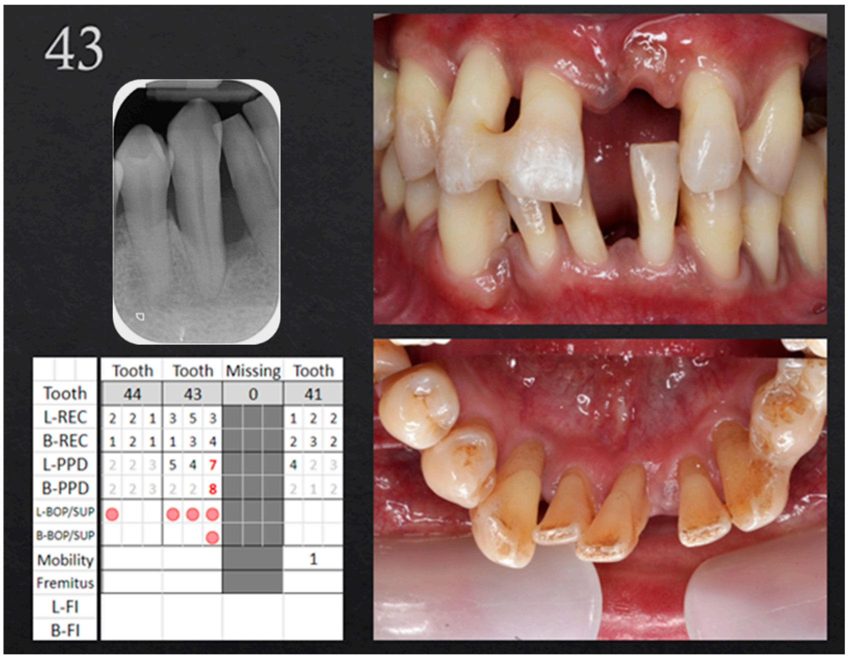

	Tooth			Tooth			Missing			Tooth		
Tooth	44			43			0			41		
L-REC	2	2	1	3	5	3				1	2	2
B-REC	1	2	1	1	3	4				2	3	2
L-PPD	2	2	3	5	4	7				4	2	3
B-PPD	2	2	3	2	2	8				2	1	2
L-BOP/SUP	●			●	●	●						
B-BOP/SUP						●						
Mobility											1	
Fremitus												
L-FI												
B-FI												

The periapical radiograph is scaled to actual size.

Please enter the start time for this case questionnaire at the top left corner.

1. **Are you currently a:**
   ☐ Second-year resident
   ☐ Third-year resident
   ☐ Resident in higher training
   ☐ Specialist

Please find a fixed CBCT axial view of the defect below. Each slice is of 0.2mm thickness. The interval between each slice is 0.5mm. Please note that the slices are magnified for ease of viewing.

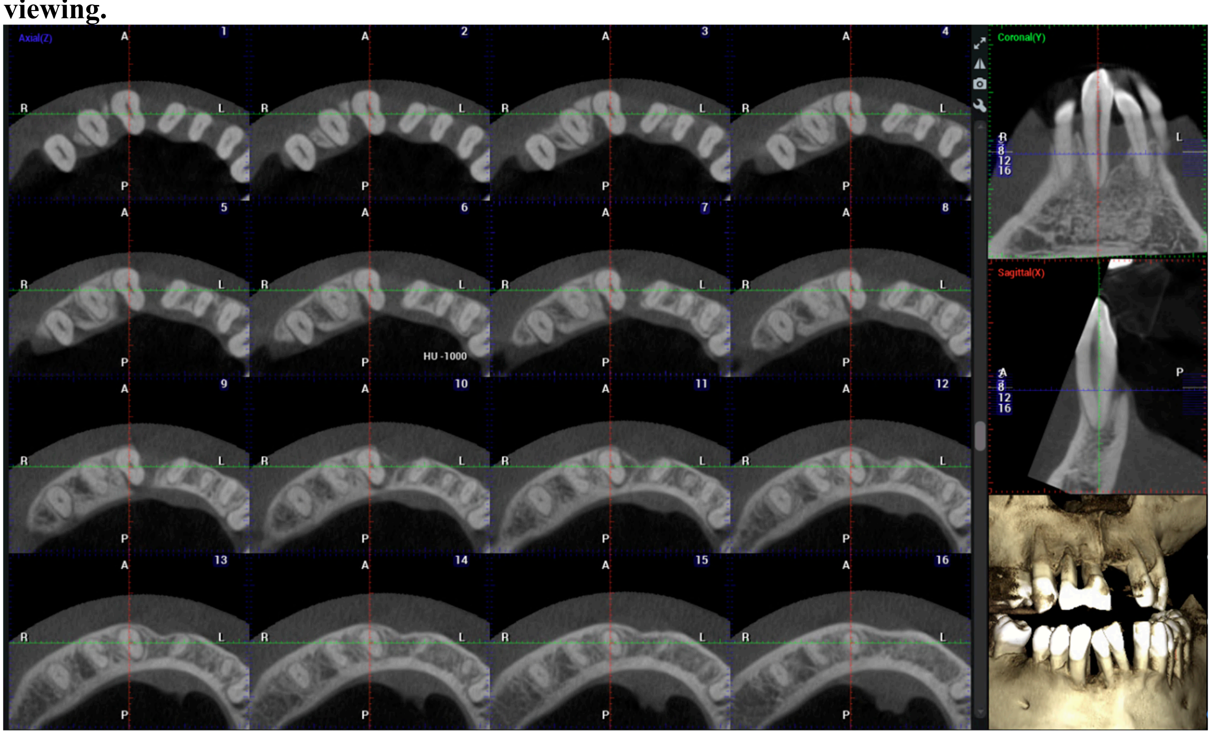

2. **Does the defect involve:**
   - **a.** More than 1 surface of the root of the same tooth? ☐ Yes ☐ No
     [If you answered "No" for a), please proceed to Q3]
   - **b.** If you answered "Yes" for a), which surfaces of the root does the defect involve? You may choose more than one answer:
     - ☐ Mesial and buccal
     - ☐ Mesial and lingual
     - ☐ Mesial - buccal - distal
     - ☐ Mesial - lingual - distal
     - ☐ Circumferential

3. **Is there a presence of the following type(s) of infrabony defect? You may choose more than one answer:**
   - ☐ 1-walled component
   - ☐ 2-walled component
   - ☐ 3-walled component
   - ☐ Crater
   - ☐ Circumferential defect

4. **Is the depth of the defect ≥ 3mm?**
   [Defect depth = (number of axial slices involving the defect -1) x 0.5mm + (0.2mm / 2) x 2]
   ☐ Yes ☐ No

Please find a fixed CBCT sagittal view of the defect below. The slice is magnified for ease of viewing.

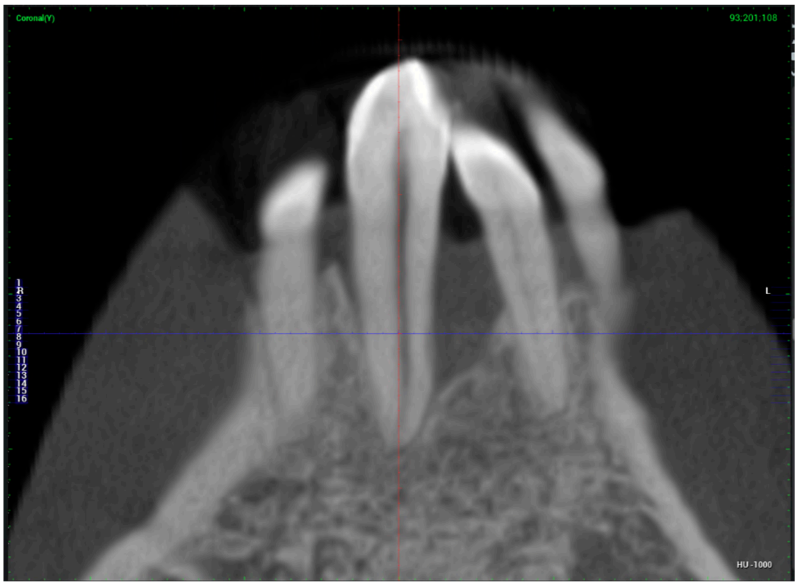

5. **Is the angle of the defect ≤ 22 degrees?**
   ☐ Yes ☐ No

6. **Is the angle of the defect ≥ 36 degrees?**
   ☐ Yes ☐ No

7. **Simply considering the defect presentation alone, which procedure do you think is the most appropriate for site management? Please choose only one option:**
   - ☐ Non-surgical maintenance
   - ☐ Access flap
   - ☐ Access flap with osseous resective surgery
   - ☐ Periodontal regeneration
   - ☐ Tooth extraction
   - ☐ Others (Please specify: ____)

8. **If periodontal regeneration is selected for Question 7:**
   a. **Which regenerative approach would you select? Please choose only one option:**
      - ☐ Modified minimally invasive surgical technique (M-MIST)
      - ☐ Minimally invasive surgical technique (MIST)
      - ☐ Single flap approach (SFA)
      - ☐ Others (Please specify: ____)

   b. **What biomaterials would you consider using? Please choose only one option:**
      - ☐ EMD only
      - ☐ Bone graft only
      - ☐ Resorbable membrane only
      - ☐ EMD + bone graft
      - ☐ Bone graft + resorbable membrane
      - ☐ EMD + bone graft + resorbable membrane
      - ☐ No biomaterial
      - ☐ Others (Please specify: ____)

End time: ________________

Start time: ________________

14 mesial

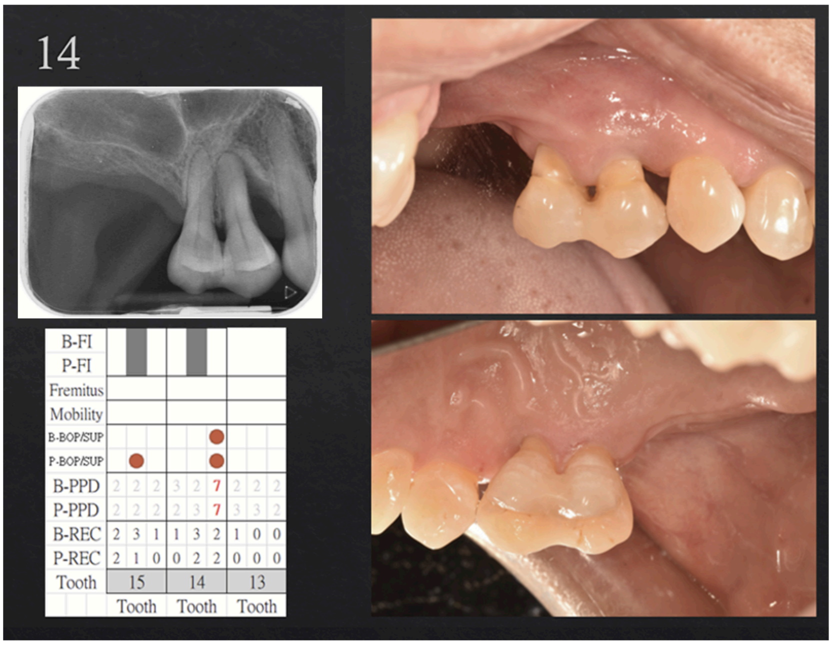

The periapical radiograph is scaled to actual size.

Please enter the start time for this case questionnaire at the top left corner.

1. **Are you currently a:**
   ☐ Second-year resident
   ☐ Third-year resident
   ☐ Resident in higher training
   ☐ Specialist

Please find a fixed CBCT axial view of the defect below. Each slice is of 0.2mm thickness. The interval between each slice is 0.5mm. Please note that the slices are magnified for ease of viewing.

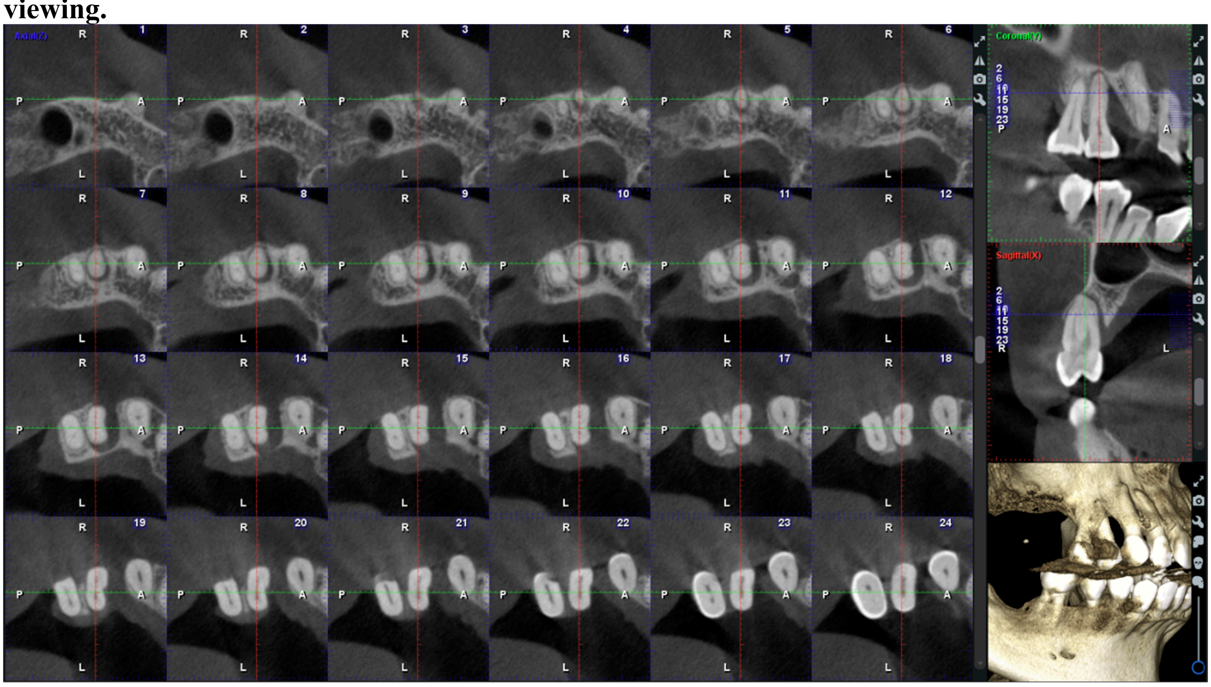

2. **Does the defect involve:**
   a. More than 1 surface of the root of the same tooth? ☐ Yes ☐ No
   [If you answered "No" for a), please proceed to Q3]
   b. If you answered "Yes" for a), which surfaces of the root does the defect involve? You may choose more than one answer:
   ☐ Mesial and buccal
   ☐ Mesial and palatal
   ☐ Mesial - buccal - distal
   ☐ Mesial - palatal - distal
   ☐ Circumferential

3. **Is there a presence of the following type(s) of infrabony defect? You may choose more than one answer:**
   ☐ 1-walled component
   ☐ 2-walled component
   ☐ 3-walled component
   ☐ Crater
   ☐ Circumferential defect

4. **Is the depth of the defect ≥ 3mm?**
   [Defect depth = (number of axial slices involving the defect -1) x 0.5mm + (0.2mm / 2) x 2]
   ☐ Yes ☐ No

Please find a fixed CBCT sagittal view of the defect below. The slice is magnified for ease of viewing.

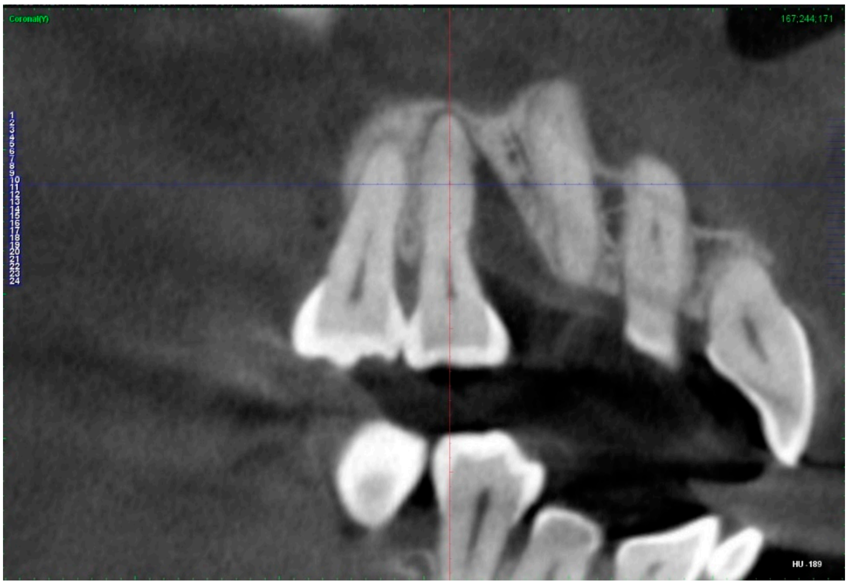

5. **Is the angle of the defect ≤ 22 degrees?**
   ☐ Yes ☐ No

6. **Is the angle of the defect ≥ 36 degrees?**
   ☐ Yes ☐ No

7. **Simply considering the defect presentation alone, which procedure do you think is the most appropriate for site management? Please choose only one option:**
   ☐ Non-surgical maintenance
   ☐ Access flap
   ☐ Access flap with osseous resective surgery
   ☐ Periodontal regeneration
   ☐ Tooth extraction
   ☐ Others (Please specify:____)

8. **If periodontal regeneration is selected for Question 7:**
   a. **Which regenerative approach would you select? Please choose only one option:**
      ☐ Modified minimally invasive surgical technique (M-MIST)
      ☐ Minimally invasive surgical technique (MIST)
      ☐ Single flap approach (SFA)
      ☐ Others (Please specify: ____)
   b. **What biomaterials would you consider using? Please choose only one option:**
      ☐ EMD only
      ☐ Bone graft only
      ☐ Resorbable membrane only
      ☐ EMD + bone graft
      ☐ Bone graft + resorbable membrane
      ☐ EMD + bone graft + resorbable membrane
      ☐ No biomaterial
      ☐ Others (Please specify: ____)

End time: ________________

Start time: ________________

43 distal

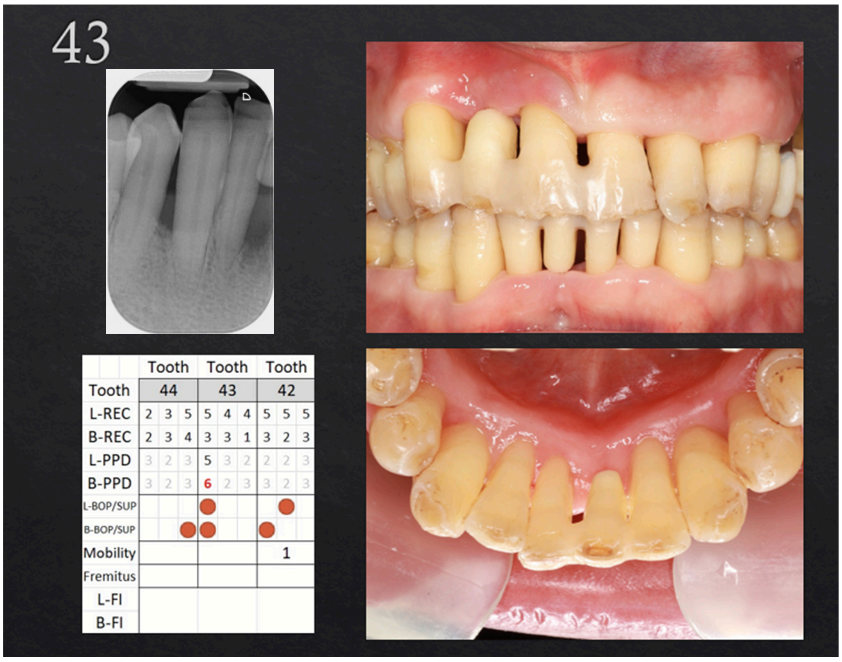

	Tooth			Tooth			Tooth		
Tooth	44			43			42		
L-REC	2	3	5	5	4	4	5	5	5
B-REC	2	3	4	3	3	1	3	2	3
L-PPD	3	2	3	5	3	2	2	2	3
B-PPD	3	2	3	6	2	3	3	2	3
L-BOP/SUP				●				●	
B-BOP/SUP			●	●			●		
Mobility								1	
Fremitus									
L-FI									
B-FI									

The periapical radiograph is scaled to actual size.

Please enter the start time for this case questionnaire at the top left corner.

1. **Are you currently a:**
   ☐ Second-year resident
   ☐ Third-year resident
   ☐ Resident in higher training
   ☐ Specialist

Please find a fixed CBCT axial view of the defect below. Each slice is of 0.2mm thickness. The interval between each slice is 0.5mm. Please note that the slices are magnified for ease of viewing.

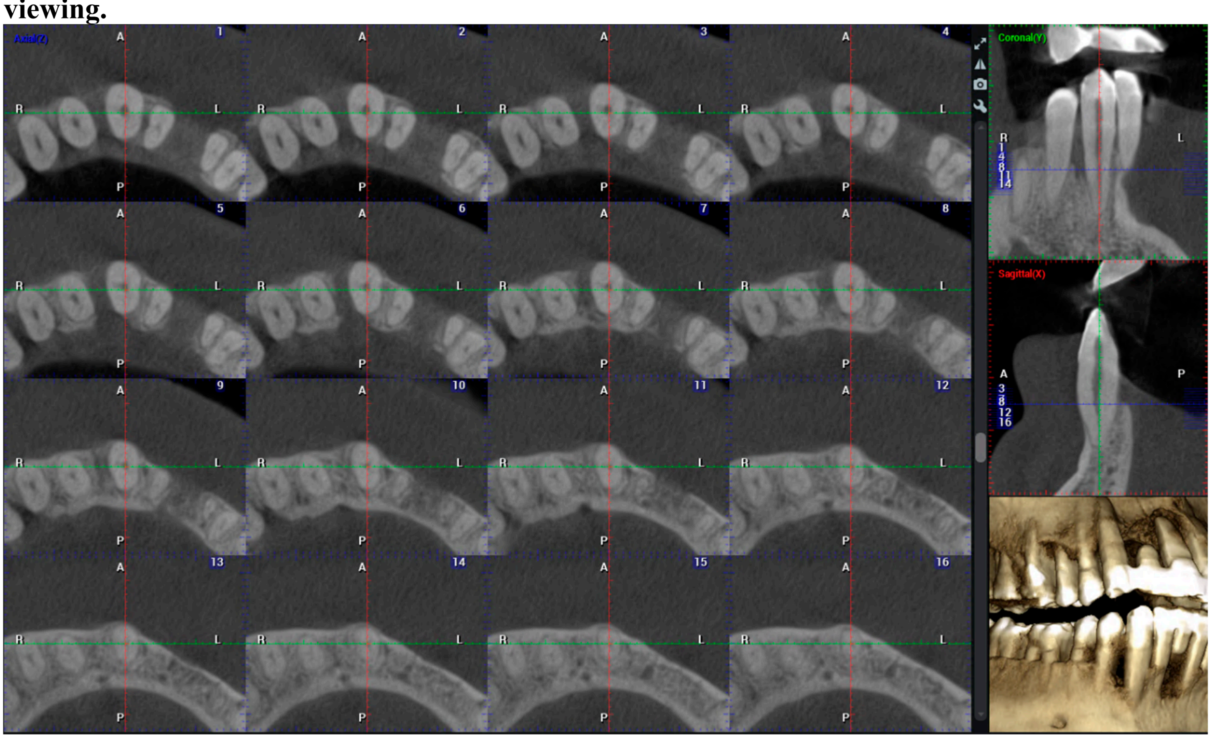

2. **Does the defect involve:**
   a. More than 1 surface of the root of the same tooth? ☐ Yes ☐ No
   [If you answered "No" for a), please proceed to Q3]
   b. If you answered "Yes" for a), which surfaces of the root does the defect involve? You may choose more than one answer:
      ☐ Distal and buccal
      ☐ Distal and lingual
      ☐ Distal - buccal - mesial
      ☐ Distal - lingual - mesial
      ☐ Circumferential

3. **Is there a presence of the following type(s) of infrabony defect? You may choose more than one answer:**
   ☐ 1-walled component
   ☐ 2-walled component
   ☐ 3-walled component
   ☐ Crater
   ☐ Circumferential defect

4. **Is the depth of the defect ≥ 3mm?**
   [Defect depth = (number of axial slices involving the defect -1) x 0.5mm + (0.2mm / 2) x 2]
   ☐ Yes ☐ No

Please find a fixed CBCT sagittal view of the defect below. The slice is magnified for ease of viewing.

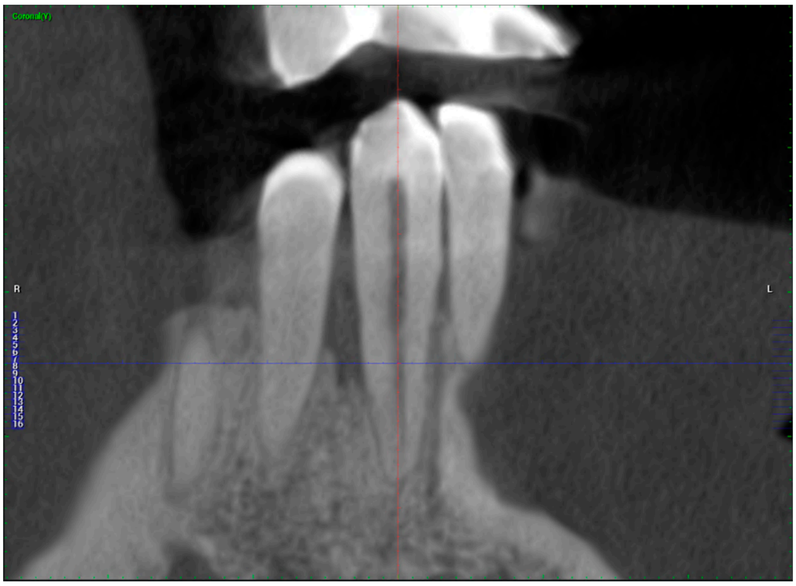

5. **Is the angle of the defect ≤ 22 degrees?**
   ☐ Yes ☐ No

6. **Is the angle of the defect ≥ 36 degrees?**
   ☐ Yes ☐ No

7. **Simply considering the defect presentation alone, which procedure do you think is the most appropriate for site management? Please choose only one option:**
   - ☐ Non-surgical maintenance
   - ☐ Access flap
   - ☐ Access flap with osseous resective surgery
   - ☐ Periodontal regeneration
   - ☐ Tooth extraction
   - ☐ Others (Please specify: ____)

8. **If periodontal regeneration is selected for Question 7:**
   a. **Which regenerative approach would you select? Please choose only one option:**
      - ☐ Modified minimally invasive surgical technique (M-MIST)
      - ☐ Minimally invasive surgical technique (MIST)
      - ☐ Single flap approach (SFA)
      - ☐ Others (Please specify: ____)

   b. **What biomaterials would you consider using? Please choose only one option:**
      - ☐ EMD only
      - ☐ Bone graft only
      - ☐ Resorbable membrane only
      - ☐ EMD + bone graft
      - ☐ Bone graft + resorbable membrane
      - ☐ EMD + bone graft + resorbable membrane
      - ☐ No biomaterial
      - ☐ Others (Please specify: ____)

End time: ________________

Start time: ________________

41 distal

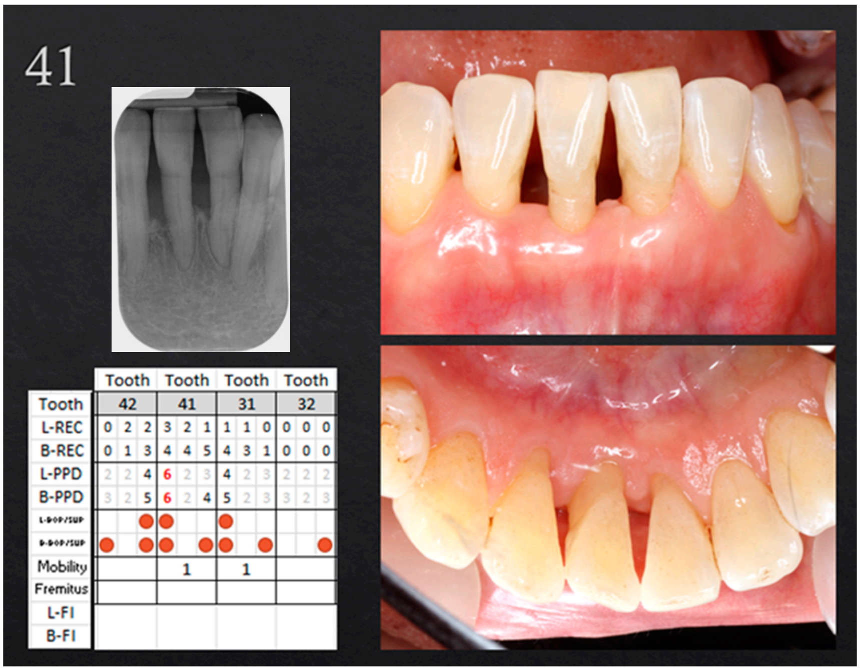

	Tooth			Tooth			Tooth			Tooth		
Tooth	42			41			31			32		
L-REC	0	2	2	3	2	1	1	1	0	0	0	0
B-REC	0	1	3	4	4	5	4	3	1	0	0	0
L-PPD	2	2	4	6	2	3	4	2	3	2	2	2
B-PPD	3	2	5	6	2	4	5	2	3	3	2	3
L-BOP/SUP			●	●			●					
B-BOP/SUP	●		●	●		●	●		●			●
Mobility					1			1				
Fremitus												
L-FI												
B-FI												

The periapical radiograph is scaled to actual size.

Please enter the start time for this case questionnaire at the top left corner.

1. **Are you currently a:**
   ☐ Second-year resident
   ☐ Third-year resident
   ☐ Resident in higher training
   ☐ Specialist

Please find a fixed CBCT axial view of the defect below. Each slice is of 0.2mm thickness. The interval between each slice is 0.5mm. Please note that the slices are magnified for ease of viewing.

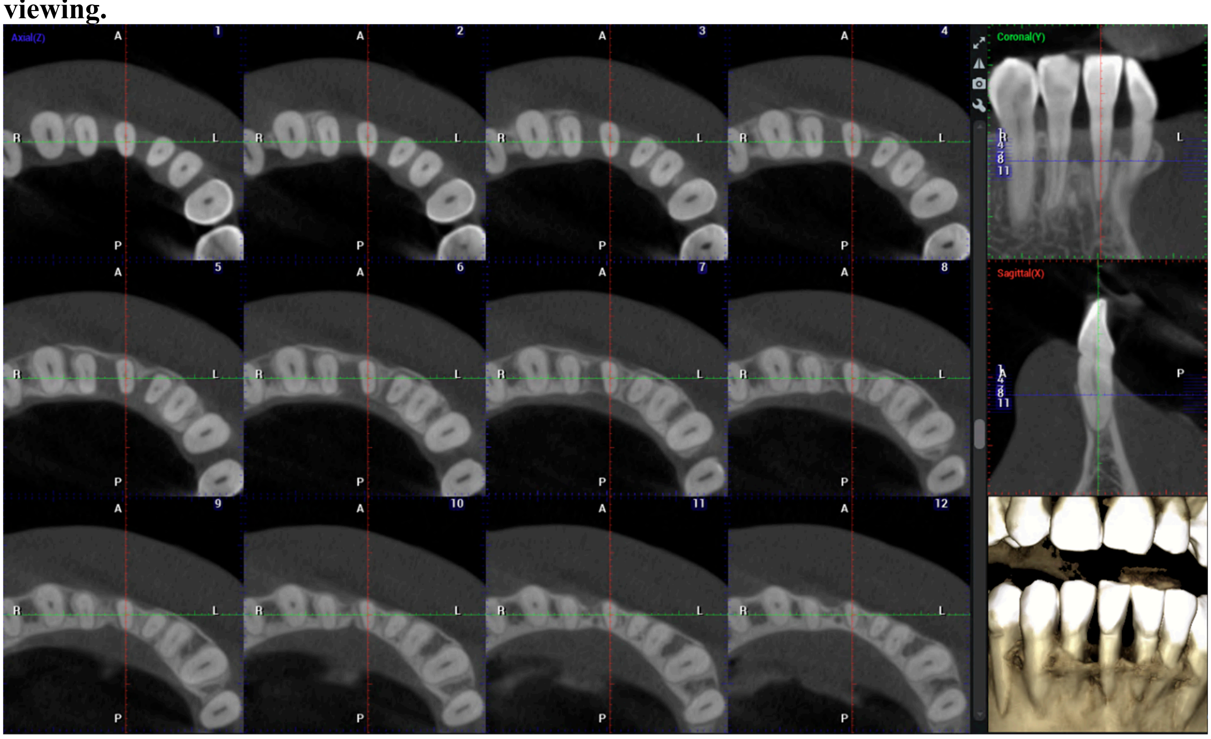

2. **Does the defect involve:**
   - **a.** More than 1 surface of the root of the same tooth? ☐ Yes ☐ No
     [If you answered "No" for a), please proceed to Q3]
   - **b.** If you answered "Yes" for a), which surfaces of the root does the defect involve? You may choose more than one answer:
     - ☐ Distal and buccal
     - ☐ Distal and lingual
     - ☐ Distal - buccal - mesial
     - ☐ Distal - lingual - mesial
     - ☐ Circumferential

3. **Is there a presence of the following type(s) of infrabony defect? You may choose more than one answer:**
   ☐ 1-walled component
   ☐ 2-walled component
   ☐ 3-walled component
   ☐ Crater
   ☐ Circumferential defect

4. **Is the depth of the defect ≥ 3mm?**
   [Defect depth = (number of axial slices involving the defect -1) x 0.5mm + (0.2mm / 2) x 2]
   ☐ Yes ☐ No

Please find a fixed CBCT sagittal view of the defect below. The slice is magnified for ease of viewing.

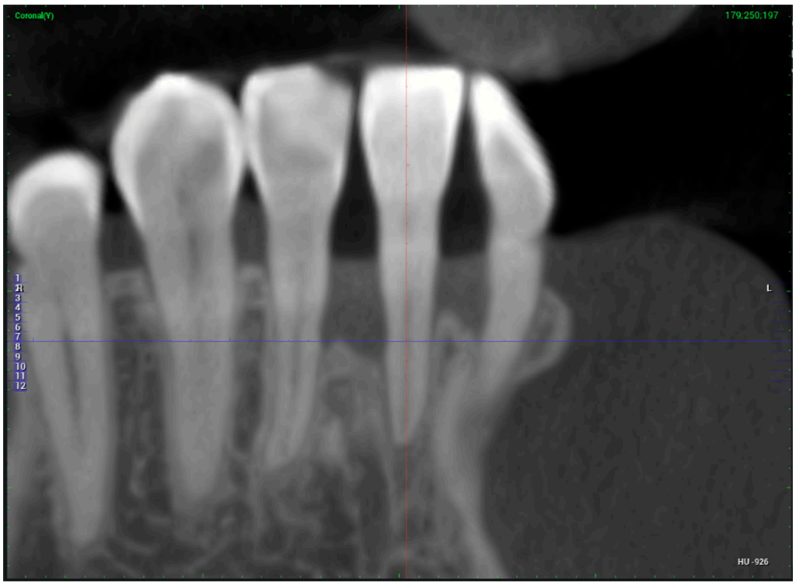

5. **Is the angle of the defect ≤ 22 degrees?**
   ☐ Yes ☐ No

6. **Is the angle of the defect ≥ 36 degrees?**
   ☐ Yes ☐ No

7. **Simply considering the defect presentation alone, which procedure do you think is the most appropriate for site management? Please choose only one option:**
   ☐ Non-surgical maintenance
   ☐ Access flap
   ☐ Access flap with osseous resective surgery
   ☐ Periodontal regeneration
   ☐ Tooth extraction
   ☐ Others (Please specify: ____)

8. **If periodontal regeneration is selected for Question 7:**
   a. **Which regenerative approach would you select? Please choose only one option:**
      ☐ Modified minimally invasive surgical technique (M-MIST)
      ☐ Minimally invasive surgical technique (MIST)
      ☐ Single flap approach (SFA)
      ☐ Others (Please specify: ____)

   b. **What biomaterials would you consider using? Please choose only one option:**
      ☐ EMD only
      ☐ Bone graft only
      ☐ Resorbable membrane only
      ☐ EMD + bone graft
      ☐ Bone graft + resorbable membrane
      ☐ EMD + bone graft + resorbable membrane
      ☐ No biomaterial
      ☐ Others (Please specify: ____)

End time: ________________

Start time: ________________

16 mesial

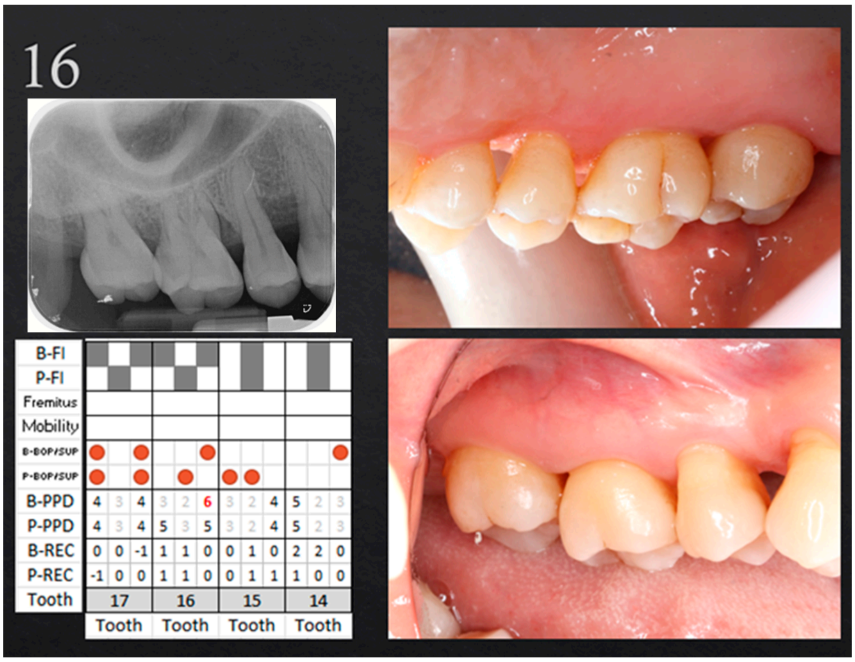

The periapical radiograph is scaled to actual size.

Please enter the start time for this case questionnaire at the top left corner.

1. **Are you currently a:**
   ☐ Second-year resident
   ☐ Third-year resident
   ☐ Resident in higher training
   ☐ Specialist

Please find a fixed CBCT axial view of the defect below. Each slice is of 0.2mm thickness. The interval between each slice is 0.5mm. Please note that the slices are magnified for ease of viewing.

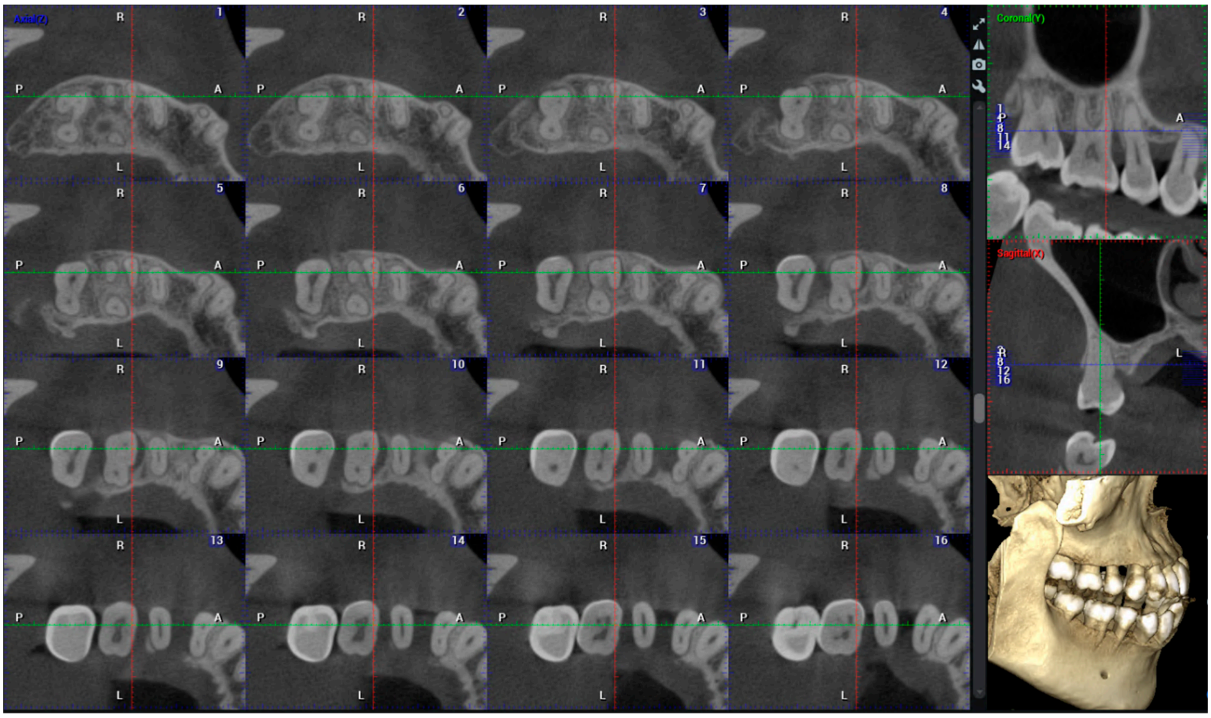

2. **Does the defect involve:**
   a. More than 1 surface of the root of the same tooth? ☐ Yes ☐ No
      [If you answered "No" for a), please proceed to Q3]
   b. If you answered "Yes" for a), which surfaces of the root does the defect involve? You may choose more than one answer:
      - ☐ Mesial and buccal
      - ☐ Mesial and palatal
      - ☐ Mesial - buccal - distal
      - ☐ Mesial - palatal - distal
      - ☐ Circumferential

3. **Is there a presence of the following type(s) of infrabony defect? You may choose more than one answer:**
   - ☐ 1-walled component
   - ☐ 2-walled component
   - ☐ 3-walled component
   - ☐ Crater
   - ☐ Circumferential defect

4. **Is the depth of the defect ≥ 3mm?**
   [Defect depth = (number of axial slices involving the defect -1) x 0.5mm + (0.2mm / 2) x 2]
   ☐ Yes ☐ No

Please find a fixed CBCT sagittal view of the defect below. The slice is magnified for ease of viewing.

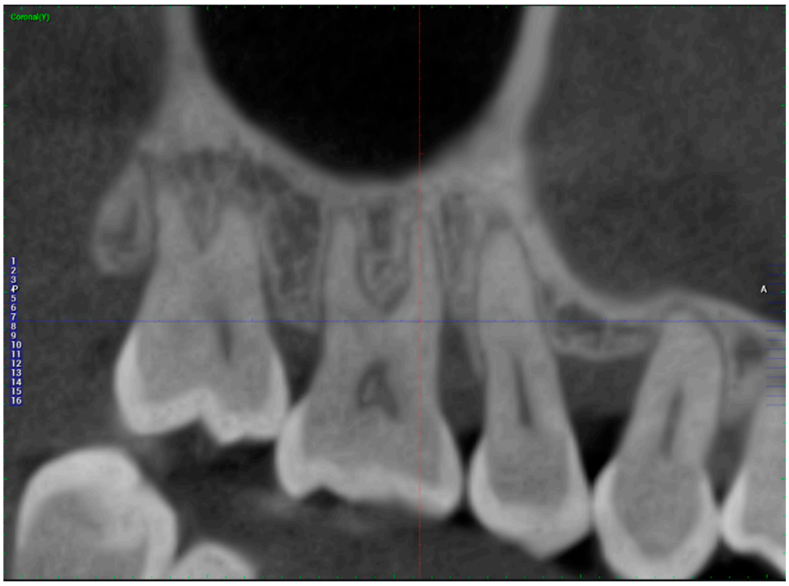

5. **Is the angle of the defect ≤ 22 degrees?**
   ☐ Yes ☐ No

6. **Is the angle of the defect ≥ 36 degrees?**
   ☐ Yes ☐ No

7. **Simply considering the defect presentation alone, which procedure do you think is the most appropriate for site management? Please choose only one option:**
   ☐ Non-surgical maintenance
   ☐ Access flap
   ☐ Access flap with osseous resective surgery
   ☐ Periodontal regeneration
   ☐ Tooth extraction
   ☐ Others (Please specify: ______________________________)

8. **If periodontal regeneration is selected for Question 7:**
   a. **Which regenerative approach would you select? Please choose only one option:**
      ☐ Modified minimally invasive surgical technique (M-MIST)
      ☐ Minimally invasive surgical technique (MIST)
      ☐ Single flap approach (SFA)
      ☐ Others (Please specify: ______________________________)
   b. **What biomaterials would you consider using? Please choose only one option:**
      ☐ EMD only
      ☐ Bone graft only
      ☐ Resorbable membrane only
      ☐ EMD + bone graft
      ☐ Bone graft + resorbable membrane
      ☐ EMD + bone graft + resorbable membrane
      ☐ No biomaterial
      ☐ Others (Please specify: ______________________________)

End time: ______________

Supplementary Table S1

Intra-oral radiograph exposure values used with short cone

	Incisors			Canines and Premolars			Molars		
	**kV**	**mA**	**time (s)**	**kV**	**mA**	**time (s)**	**kV**	**mA**	**time (s)**
**Maxilla**	60	7	0.25	60	7	0.32	60	7	0.40
**Mandible**	60	7	0.16	60	7	0.20	60	7	0.32
